# Vascular smooth muscle cell-derived osteoprotegerin drives pulmonary arterial hypertension via AKT activation and is attenuated by monoclonal antibody therapy

**DOI:** 10.1093/cvr/cvag197

**Published:** 2026-09-07

**Authors:** Merve Keles, Laura E West, Nadine D Arnold, Astrid Weiss, Nadiya V Mykytyuk, Julia Baldauf, Josephine A Pickworth, Amira A Zawia, Helena A Turton, Megan F Allen, Clemens Ruppert, Alfred A Roger Thompson, Sheila E Francis, Ralph T Schermuly, Allan Lawrie

**Affiliations:** National Heart and Lung Institute, Faculty of Medicine, Imperial College London, London W12 0NN, UK; School of Medicine and Population Health, University of Sheffield, Sheffield, UK; School of Medicine and Population Health, University of Sheffield, Sheffield, UK; Department of Internal Medicine, German Center for Lung Research (DZL), Excellence Cluster Cardio-Pulmonary Institute (CPI), Institute for Lung Health (ILH), Justus Liebig University, Giessen, Germany; National Heart and Lung Institute, Faculty of Medicine, Imperial College London, London W12 0NN, UK; Department of Internal Medicine, German Center for Lung Research (DZL), Excellence Cluster Cardio-Pulmonary Institute (CPI), Institute for Lung Health (ILH), Justus Liebig University, Giessen, Germany; School of Medicine and Population Health, University of Sheffield, Sheffield, UK; School of Medicine and Population Health, University of Sheffield, Sheffield, UK; School of Medicine and Population Health, University of Sheffield, Sheffield, UK; School of Medicine and Population Health, University of Sheffield, Sheffield, UK; Department of Internal Medicine, German Center for Lung Research (DZL), Excellence Cluster Cardio-Pulmonary Institute (CPI), Institute for Lung Health (ILH), Justus Liebig University, Giessen, Germany; UGMLC/DZL Giessen Biobank, Justus Liebig University, Giessen, Germany; School of Medicine and Population Health, University of Sheffield, Sheffield, UK; School of Medicine and Population Health, University of Sheffield, Sheffield, UK; Department of Internal Medicine, German Center for Lung Research (DZL), Excellence Cluster Cardio-Pulmonary Institute (CPI), Institute for Lung Health (ILH), Justus Liebig University, Giessen, Germany; National Heart and Lung Institute, Faculty of Medicine, Imperial College London, London W12 0NN, UK

**Keywords:** Osteoprotegerin, Pulmonary hypertension, Vascular smooth muscle cell, Vascular remodelling

## Abstract

**Aims:**

Pulmonary arterial hypertension (PAH) is driven by sustained vasoconstriction and structural remodelling of distal pulmonary arteries. We have previously demonstrated that osteoprotegerin (OPG, *tnfrsf11b*) is upregulated in PAH animal models and patient tissues, with circulating levels being prognostic. OPG knock-out (KO) mice exhibit an attenuated PAH phenotype, and therapeutic inhibition using a human monoclonal anti-OPG antibody reduces disease severity in multiple rodent models. However, the key cellular source of pathogenic OPG and the mechanism underlying therapeutic blockade remain unknown.

**Methods and results:**

To define the main cellular source of OPG, conditional *Tnfrsf11b* deletion was generated by crossing OPG^fl/fl^ mice with PGKcre^tg/+^ (global), LysMcre^tg/+^ (myeloid), Myl2cre^tg/+^ (cardiac myocytes), Cdh5cre^tg/+^ (endothelial cells), smMHCcre^tg/+^ [vascular smooth muscle cell (VSMCs)], Col1a2cre^tg/+^ (fibroblasts). KOs were induced by tamoxifen injection, and PAH established using Sugen5416/hypoxia (SuHx) model. Cardiopulmonary phenotyping included echocardiography, cardiac catheterization, and histological analysis. Mechanistic studies were performed in VSMCs using peptide-based kinase activity profiling (PamStation).

PGKcre^tg/+^/OPG^fl/fl^ mice phenocopied the previous results obtained with global OPG KO (OPG^−/−^). Only VSMC-specific deletion (smMHCcre^tg/0^/OPG^fl/fl^) resulted in significantly improved haemodynamic parameters, evidenced by reduced right ventricular systolic pressure and pulmonary vascular resistance, and reduced pulmonary vascular remodelling demonstrating that VSMC-derived OPG is the primary driver of PAH pathogenesis.

Kinome profiling of control and idiopathic PAH (IPAH)-pulmonary artery smooth muscle cells (PASMCs) treated with OPG^+/−^ anti-OPG antibody revealed IPAH-specific regulation of PI3K–Akt and enrichment of pathways involving autophagy, and longevity regulation linked to cellular stress resistance and growth. Anti-OPG antibody treatment reduced AKT phosphorylation in IPAH-PASMCs. Knock-down of AKT1 by siRNA reduced OPG-induced proliferation and migration of human PASMCs. These results align with emerging data implicating OPG in cellular senescence and vascular ageing.

**Conclusion:**

Together, these findings highlight VSMC-derived OPG as a critical mediator of pulmonary vascular remodelling in PAH pathogenesis and suggest that targeting OPG via a therapeutic antibody is mediated through PI3K–Akt signalling.

## Graphical Abstract

**Graphical Abstract:**
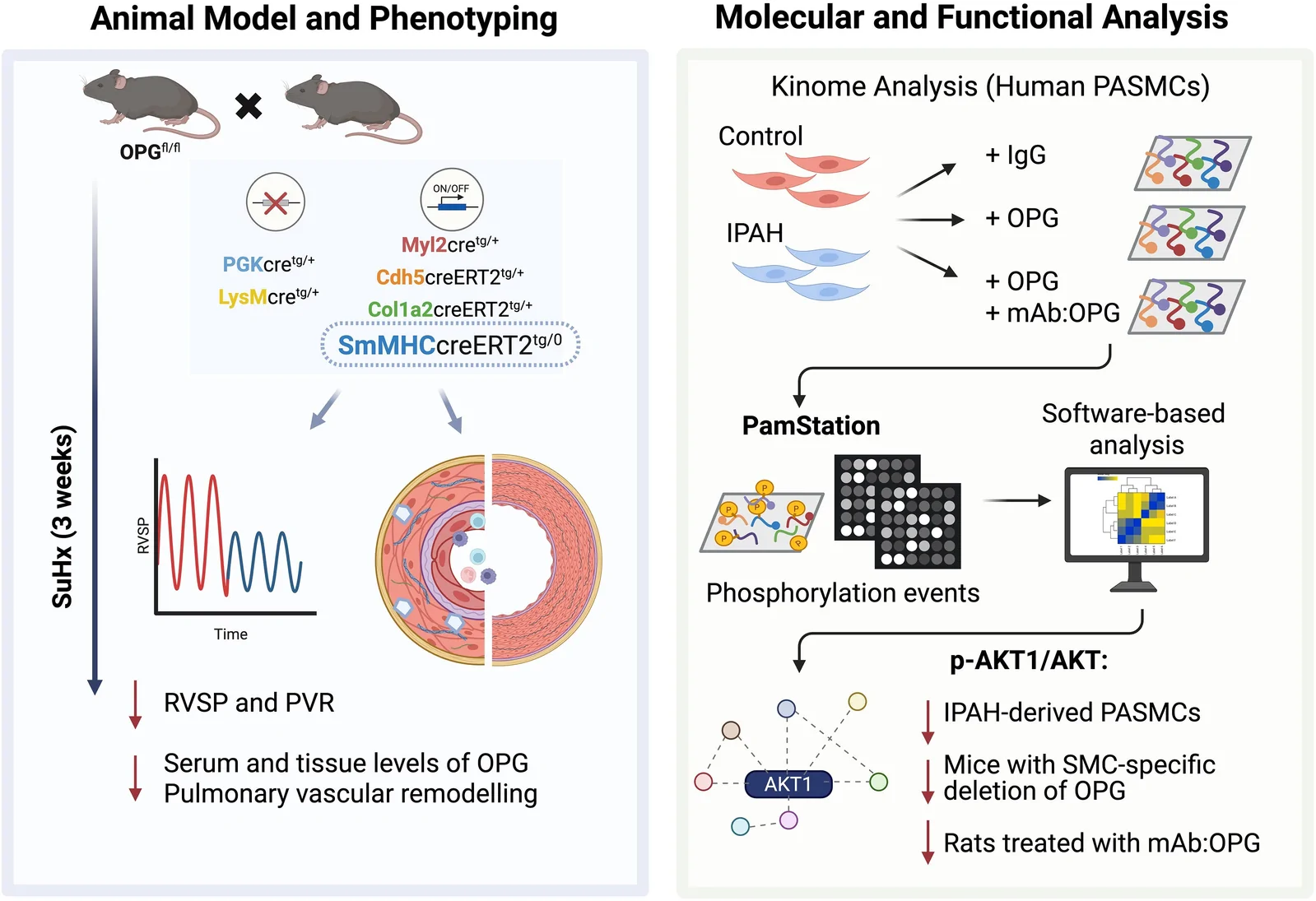
Left panel: schematic shows the generation of cell-type specific OPG knock-out mice, phenotyping process and desired outputs. Only smooth muscle cell deletion of OPG resulted in reduced remodelling and PAH phenotype. Right panel: schematic shows the molecular analysis of downstream OPG signalling in PASMC and highlights p-AKT-1 as the driver of OPG induced vascular changes. Created in BioRender. Keles, M. (2026) https://BioRender.com/7v1p3zs.

**Time of primary review: 55 days**

## 1. Introduction

Pulmonary arterial hypertension (PAH) is a severe disease characterized by both sustained vasoconstriction and progressive obliteration of small (distal) pulmonary arteries through medial thickening, intimal fibrosis, and the formation of angioproliferative lesions.^1,2^ This remodelling of the pulmonary vasculature leads to increased resistance and elevated pulmonary artery (PA) pressure, eventually resulting in right ventricular (RV)-mediated heart failure.^3^

Current treatments primarily target sustained vasoconstriction to relieve symptoms and improve survival;^4,5^ however, they do not halt the disease progression or reverse vascular pathology. Over the last decade, considerable advancements have been made in understanding the molecular and cellular mechanisms involved in PAH pathogenesis. These include dysregulation of bone morphogenetic protein and transforming growth factor-beta signalling pathways,^6^ and the involvement of growth factors such as platelet-derived growth factor.^7^ This has led to the development of potential disease modifying therapies such as sotatercept^8^ and imatinib.^9,10^ However, further research is needed to fully understand the long-term effects of these drugs on PAH pathogenesis to evaluate their sustained efficacy and safety.^11^ These limitations underscore the need to explore additional therapeutic targets.

Pathologically, PAH is characterized by dysregulated pulmonary arterial endothelial cells, which exhibit increased proliferation and resistance to apoptosis.^12–14^ Subsequent proliferation and migration of PA smooth muscle cells (PASMCs) and fibroblasts (FBs), along with the recruitment of monocytes/macrophages further exacerbate vascular remodelling.^14^

Osteoprotegerin (OPG, *tnfrsf11b*) is a secreted glycoprotein within the tumour necrosis factor (TNF) receptor superfamily, most recognized for its role in bone metabolism as a decoy receptor for receptor activator of nuclear factor-κB ligand (RANKL), where it blocks RANK-mediated osteoclast differentiation and osteoclastogenesis.^15,16^ OPG has been implicated in vascular ageing and stiffness, with studies highlighting its expression in vascular smooth muscle cells (VSMCs) and its association with arterial calcification,^17,18^ senescence, and vascular remodelling.^19^ Beyond RANKL, OPG also binds to TNF-related apoptosis-inducing ligand (TRAIL, *tnfsf10*), a key regulator of apoptosis with implications in PAH preclinical models.^20–22^ In PAH, we have shown that OPG is upregulated in remodelled pulmonary vascular lesions^23^ and serum^24^ of patients with idiopathic PAH (IPAH), with elevated serum levels being prognostic. Additionally, higher OPG levels were associated with haemodynamic severity and poorer survival outcomes. Both OPG and TRAIL can drive PASMC proliferation and migration,^20,23^ and levels of OPG are elevated in both preclinical models PAH^20,25^ and within the lungs and serum of patients with IPAH.^21–24^ We have previously demonstrated that deletion or inhibition of either TRAIL^20^ or OPG^25^ attenuates and reverses PAH progression in preclinical models. Using bone-marrow-transplanted chimeric *Tnfrsf11b^−/−^* mice, we identified bone-marrow-derived OPG as the main source of OPG promoting PAH in the murine SuHx model.^25^ We identified that in human PA-SMC, OPG binds to FAS receptor (*tnfrsf6*) contributing to a pro-migratory, pro-proliferative response, increasing TRAIL expression and PASMC proliferation.^25^ However, since OPG and FAS are broadly expressed by multiple cell types, and sub-lethal irradiation approaches can induce pulmonary endothelial damage, inflammation, and altered immune cell reconstitution,^26,27^ we generated a new conditional knock-out (KO) *Tnfrsf11b* mouse to address these limitations and determine the cellular source of OPG responsible for pulmonary vascular and RV remodelling. We generated mice with global (PGK) and lineage-specific deletion of *Opg* (*Tnfrsf11b*^−/−^) in myeloid cells (LysM), ECs (Chd5-cre), FBs (Col1a2-cre), cardiomyocytes (CMs, Myl2-cre), and VSMCs (smMHC-cre). While most conditional KOs showed limited or no impact on disease phenotype, VSMC-specific OPG deletion robustly attenuated PAH, improving both haemodynamics and pulmonary vascular remodelling. To further explore the underlying mechanism, we performed kinome profiling in control and IPAH-PASMCs treated with OPG in the presence and absence of an anti-OPG antibody. This identified IPAH-specific regulation of the PI3K–Akt pathway and enrichment of signalling networks involved in autophagy, cellular stress resistance, and longevity regulation.

## 2. Methods

### 2.1 Animal models and studies

All animal studies were approved by the University of Sheffield Animal Welfare and Ethical Review Body and conducted under the authority of a UK Home Office project licence, in accordance with the UK Animals (Scientific Procedures) Act 1986 and Directive 2010/63/EU of the European Parliament on the protection of animals used for scientific purposes. Animals were provided unrestricted access to water and a standard diet, housed under a 12 h light/dark cycle at a controlled room temperature of 22 ± 2°C and a humidity of 35–60%. Investigators remained blinded to mouse genotype and treatment throughout all catheterization, echocardiography, histological assessments, and immunofluorescence (IF) quantifications.

#### 2.1.1 Generation of *Tnfrsf11b* KO mouse line

To create conditional and constitutive KO alleles of the *Tnfrsf11b* gene, a targeted strategy was applied based on the NCBI transcript NM_008764.3, located on mouse chromosome 15. Exon 1, which contains the translation initiation codon, was left intact, while Exon 2 (approximately 1.5 kb) was flanked by loxP sites to facilitate tissue-specific deletion (*Figure 1A*). A positive selection marker (puromycin resistance, PuroR) was flanked by Flippase Recognition Target (FRT) sites and inserted into intron 1 to facilitate selection in embryonic stem (ES) cells. The *Tnfrsf11b* floxed mouse is available via http://www.infrafrontier.eu/emma/strain-search/straindetails/?q=EM:15281.

**Figure 1:**
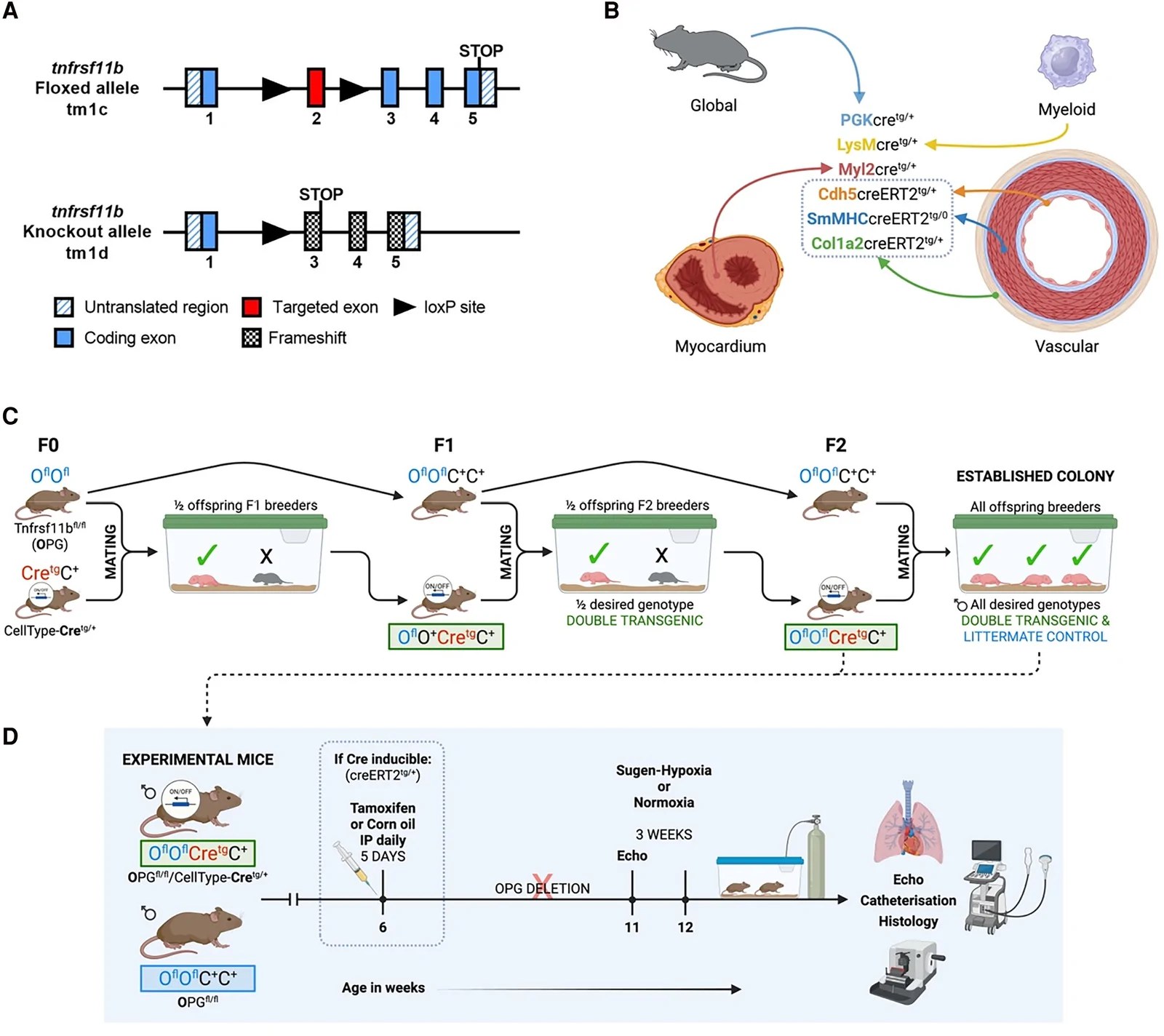
Transgenic mice breeding strategy and experimental setup for Sugen/hypoxia (SuHx) induced PAH. (*A*) Schematic representation of the targeting strategy to generate KO of the *Tnfrsf11b* gene (not drawn to scale). The upper panel showing *tnfrsf11b* allele after flp recombination, while lower panel shows the KO allele following Cre recombination. (*B*) Diagram illustrating cell-specific OPG deletion strategy which mice with floxed OPG^fl/fl^ alleles were crossed with Cre-driver lines targeting specific cell types to achieve targeted OPG deletion. Global KO (PGKcre^tg/+^), myeloid (LysMcre^tg/+^), cardiac myocytes (Myl2cre^tg/+^), ECs (Cdh5creERT2^tg/+^), VSMCs (SmMHCcreERT2^tg/0^), and FB (Col1a2creERT2^tg/+^). (*C*) Diagram illustrating the colony establishment process from the F0 to F2 generations to obtain mice with the desired genotypes. Successful breeding led to F2 offspring with cell-specific OPG KOs, which were used in PAH studies. (*D*) Schematic of the Sugen/hypoxia protocol, including tamoxifen injections for Cre-inducible KOs and corn oil injections for littermate controls. Mice received Sugen5416 injection (20 mg/kg), followed by 3 weeks of hypoxia (10% O_2_), with subsequent evaluation using echocardiography, catheterization, and histological analysis. Created in BioRender.com. A. Lawrie. (2026) https://BioRender.com/kjhu1n5.

The targeting vector was constructed using bacterial artificial chromosome (BAC) clones from the C57BL/6J RPCIB-731 BAC library. This vector was introduced into Taconic Biosciences’ C57BL/6N Tac ES cell line via electroporation. Recombinant ES cells were selected using both positive (PuroR) and negative (thymidine kinase, Tk) selection markers to confirm homologous recombination events. Correctly targeted clones were validated through polymerase chain reaction and sequencing.

Following recombination, the conditional KO allele was generated via *in vivo* Flp-mediated excision of the PuroR selection marker to produce the conditional floxed allele ‘tm1c’. The constitutive KO allele (tm1d) was subsequently produced by *in vivo* Cre-mediated deletion of Exon 2. This deletion disrupts *Tnfrsf11b* function by removing essential cysteine-rich domains, introducing a frameshift, and resulting in a premature stop codon in Exon 3 (*Figure 1A*), which likely triggers non-sense mediated RNA decay, leading to reduced gene expression. All remaining recombination sites were positioned in non-conserved genomic regions to minimize off-target effects.

#### 2.1.2 Generation of double-transgenic mouse colonies

Transgenic mouse colonies were generated by crossing the indicated mouse genotypes in the Supplementary material online, *Table S1*. Mouse colonies were consequently genotyped for floxed alleles by using the primers given in Supplementary material online, *Table S2*. Cell-specific deletion of *Tbfrsf11b* (*Opg*) was achieved by crossing OPG^fl/fl^ mice with various Cre-driver lines to generate double-transgenic animals: PGKcre^tg/+^ (ubiquitous), LysMcre^tg/+^ (myeloid cells), Myl2cre^tg/+^ (cardiac myocytes), Cdh5cre^tg/+^ (ECs), smMHCcre^tg/0^ (VSMC), and Col1a2cre^tg/+^ (FBs) (*Figure 1B*). KO of *tnfrsf6* (*Fas*) (JAX Labs, Stain no.: 026643) in VSMC was performed by crossing Fas^fl/fl^ mice with smMHCcre^tg/0^.

The inducible B6.FVB-Tg(Myh11-cre/ERT2)1Soff/J^28^ (hereafter referred to as SMMHC-Cre-ERT2), which drives tamoxifen-inducible Cre expression under control of the smooth muscle myosin heavy chain 11 (*Myh11*) promoter, was used for targeting VSMCs.^29^ Since the CreERT2 transgene is linked to Y-chromosome, only male mice were used for breeding and experiments with this transgene. Littermate control mice were generated during breeding (*Figure 1C*), with cre-negative tm1c mice, or corn oil-treated cre-positive mice used depending on the experimental cre line.

#### 2.1.3 PAH induction in mice using Sugen/hypoxia (SuHx) model

Following tamoxifen injections for Cre-inducible KOs (2 mg/mouse/day for 5 days) and corn oil injections for littermate controls, SuHx model was used to induce PAH in male mice at the age of 10–12 weeks (*Figure 1C*). PAH was induced through weekly subcutaneous injections of Sugen-5416 (Tocris, Bristol, UK) at a dose of 20 mg/kg body weight over a period of 3 weeks. Mice were maintained in hypoxic chambers, simulating an altitude of approximately 18 000 feet, for 3 weeks (Day 1–21). At the end of this period (Day 21), animals were sacrificed for endpoint procedures (*Figure 1D*).

### 2.2 PAH phenotyping

#### 2.2.1 Transthoracic echocardiography

Echocardiography was performed as described previously using a linear 30–40 MHz transducer (MX-550D) and the Vevo 3100 system (VisualSonics, Toronto, Canada). Animals were placed dorsally on a heated table to maintain the body temperature during the procedure, while ECG and respiration rates were continuously recorded. Anaesthesia was induced and maintained using isoflurane (maintenance at 0.5–1.0% after induction with 2% isoflurane) to sustain heart rates at 450–500 b.p.m. in mice.

B-mode and M-mode images were obtained in parasternal long-axis (PSLAX) and short-axis views. Left ventricular function (left ventricular internal diameter [LVID], left ventricular ejection fraction [LV-EF], left ventricular fractional shortening [LV-FS]) was quantified from PSLAX recordings. RV structure (right ventricular free-wall thickness [RVFWT], right ventricular internal diameter [RVID]) was assessed from the right PSLAX view. PA haemodynamics (peak flow, pulmonary artery acceleration time [PA AT], pulmonary artery ejection time [PA ET]) were measured using PW Doppler. Data analysis was performed in Vevo Lab (v5.5.0). See Supplementary material online, Methods for full procedural details.

#### 2.2.2 Haemodynamic measurement of right and left ventricular pressure

Left and RV catheterization was performed under isoflurane-induced anaesthesia (maintenance at 1.5% after induction with 3% isoflurane) by using a closed chest method via the right internal carotid artery and right external jugular vein as described previously. See Supplementary material online, Methods for full procedural details. Following haemodynamic measurements, animals were maintained under deep anaesthesia with isoflurane inhalation (4%), and euthanasia was performed by exsanguination with cardiac puncture followed by cervical dislocation in accordance with institutional and national ethical guidelines for animal care and use. Tissues were then harvested for the analyses described below.

### 2.3 Histological and immunohistochemistry staining

Paraffin embedded lung tissues were sectioned into 5 μm slices and stained histologically with Alcian Blue Elastic van Gieson (ABEVG). immunohistochemistry (IHC) staining was performed for α-smooth muscle actin (α-SMA). For IHC, a biotinylated secondary antibody was applied, followed by the addition of an avidin–biotin enzyme complex (Vectastain Kit, Vector Laboratories, USA) and 3,3′-diaminobenzidine tetrahydrochloride substrate. Paraffin embedded heart tissues were transversely cut in half and sectioned into 5 μm slices. IF staining was performed with wheat-germ agglutinin (WGA) and isolectin B4 (IB4) to analyse structural changes in the heart. The antibodies used in IHC and IF staining are listed in Supplementary material online, *Table S3*.

Images of stained sections were captured using a Zeiss Imager Z2 microscope with an Axiocam 506 colour camera (bright-field), and a Zeiss Axio Observer with a Hamamatsu Flash 4.0 camera (fluorescence) (Carl Zeiss, Oberkochen, Germany). Images were analysed using ZEN software 2.6 Blue edition.

### 2.4 Measurement serum and tissue OPG

Serum and lung tissue OPG levels in mice were quantified using the DuoSet ELISA kit (R&D Systems, DY459) according to the manufacturer’s instructions. Tissue OPG levels were normalized to total lung protein content, which was determined using the Pierce Micro BCA Protein Assay Kit (Thermo Scientific, 23235).

### 2.5 Myography

Pulmonary arterial segments were isolated from mice and dissected into 1–2 mm rings. These segments were mounted on a wire myograph system (DMT 620M, Danish MyoTechnology, Denmark) for isometric tension recording. Baseline tension was set to 5 mN for each arterial ring and allowed to equilibrate. To assess contractile function, segments were exposed to 50 mM potassium chloride (KCl) three times, with thorough washing between each application to ensure reproducibility and tissue viability.

For evaluation of VSMC-dependent relaxation, arterial segments were pre-contracted with 10 μM phenylephrine (PE) until a stable plateau was achieved. Subsequently, cumulative concentrations of the nitric oxide donor sodium nitroprusside (SNP; 1 × 10^−9^ to 1 × 10^−6^ M) were administered to generate concentration–response curves.

### 2.6 Cell culture

Human pulmonary arterial smooth muscle cells (HPASMCs) from healthy individuals were obtained from Lonza Ltd (CC-2581, Basel, Switzerland) while HPASMCs from IPAH patients were obtained from the Universities of Giessen and Marburg Lung Center/German Center for Lung Research (UGMLC/DZL) Giessen Biobank confirming to the principles outlined in Declaration of Helsinki. The collection and use of samples were approved by the ethics committee of Justus Liebig University (approval number: 58/15). All patients provided informed written consent for the use of biomaterials for research purposes. Individual demographic details are provided in Supplementary material online, *Table S4*. Detailed protocols of cell culture experiments can be found in Supplementary Material.

### 2.7 Protein isolation

Lung tissue protein lysates were prepared from frozen tissue with radio-immunoprecipitation assay buffer (RIPA) lysis buffer with freshly added protease and phosphatase inhibitors. Protein lysates from HPASMCs were prepared using M-PER lysis buffer (Thermo Fisher Scientific, Waltham, MA, USA) containing protease and phosphatase inhibitor cocktails (Pierce, Rockford, IL, USA). Cell lysates were then transferred into pre-cooled tubes and incubated at 4°C for 1 h and centrifuged at 16 000*×g* for 15 min. The supernatant was snap-frozen for further use and stored at −80°C. Protein quantification was performed by Bicinchoninic Acid assay (BCA) assay (Thermo Fisher Scientific, Pierce BCA Protein Assay Kit, 23225) according to manufacturer’s protocol. Protein aliquots were used for peptide-based kinase assays and western blot analysis.

### 2.8 Peptide-based kinase activity assay

The peptide-based kinase activity assay was performed using the PamStation platform (PamGene International, s-Hertogenbosch, Netherlands) to analyse the activity of 380 protein kinases (about 70% of all protein kinases), i.e. serine/threonine kinase (STK) and tyrosine kinase (TK), and analysed with Evolve 12 software, as previously described. Briefly, 2 μg (for STK) and 5 μg (for TK) of protein lysate were loaded onto the respective PamChip arrays, in the presence of reaction buffer (proprietary information of PamGene) containing 1% (w/v) bovine serum albumin, STK or TK antibody mix, and ATP. Peptide phosphorylation was detected using fluorescently labelled anti-phospho antibodies (fluorescein isothiocyanate-labelled secondary antibodies) on each microchip.

Data acquisition and analysis were performed according to the manufacturer’s instructions. Briefly, fluorescence signals were monitored using a CCD camera over multiple cycles with different exposure times. Software-based image analysis was used to assess the signal intensity, with our analysis focusing only on peptides that exhibit stable signals after filtering for quality control parameters. For each treatment group, six healthy individuals (*n* = 6) and six IPAH patients (*n* = 6) were analysed. Data were represented after logarithmic (Log_2_) transformation of the processed raw signal, and the mean of log-transformed values was used for visualization prior upstream kinase prediction.

Additional protein expression analyses for validation of the kinase activity assay were performed using Simple Western and conventional western blotting, with full protocols and antibody lists (see Supplementary material online, *Tables S5* and *S6*), and unedited images (see Supplementary material online, *Figures S6* and *S7*) were provided in the Supplementary material online, Methods.

### 2.9 Correlation analysis

To examine the association between circulating and tissue levels of OPG with RV systolic pressure (RVSP) and pulmonary vascular remodelling [medial thickness and cross-sectional area (CSA)], correlation analyses were conducted in R (version 4.4.2).

Linear regression models were fitted using the *lm()* function with ordinary least squares estimation. Model performance was evaluated using coefficients of determination (*R*^2^) and *P*-values. Monotonic relationships were additionally assessed with Spearman’s rank correlation coefficient (ρ). Scatter plots were generated with the *ggplot2* package.

### 2.10 Statistics

A sample size of at least four animals was used to provide greater than 95% power to detect a 10 mm Hg difference in RVSP with a standard deviation of 3 mm Hg with 95% confidence interval. Additional animals were studied in large group comparisons and to obtain sufficient tissue for biochemical analysis.

Data analysis and statistical testing were performed using GraphPad Prism software (version 10). Data are shown as mean ± standard error of the mean (SEM). For datasets with adequate sample size, normality (Shapiro–Wilk) and variance homogeneity (Levene/Brown–Forsythe) were assessed. For most animal comparisons, the treatment group sizes are *n* < 10, where normality tests have low power and may not reliably detect non-normality; in these cases, we followed widely used practice and applied non-parametric tests. One-way or two-way analysis of variance (ANOVA) followed by appropriate *post hoc* tests for multiple comparisons (as indicated in the figure legends) was used for statistical analysis. For comparisons between only two groups, unpaired *t*-tests were performed. *P* < 0.05 was considered statistically significant.

## 3. Results

### 3.1 Cell-specific deletion of *Opg* results in differential serum and tissue levels

OPG^fl/fl^ mice (*Figure 1A*) were crossed with Cre-driver lines, including those targeting VSMC, endothelium, myeloid cells, FBs, and CMs (*Figure 1B*) to generate conditional and constitutive tissue-specific deletion of *Tnfrsf11b* (*Figure 1C*). Following cre-activation via tamoxifen (corn oil control), double-transgenic procedural mice received baseline echocardiography prior to initiation of PAH via weekly injection of Sugen 5416 and exposure to hypoxia (10% O_2_) for 3 weeks. After 3 weeks, mice were assessed for PAH, and serum and tissues harvested for downstream analysis (*Figure 1D*). After phenotyping and tissue harvesting, tissue-specific deletion of *Opg* was illustrated by dual immunohistochemistry (IHC) (see Supplementary material online, *Figure S1*). Given that OPG is a secreted protein, its levels were additionally measured in serum and whole lung tissue to quantify the efficacy of the cell-specific KOs. Near-global deletion of OPG driven by PGK-cre resulted in an almost complete reduction in both serum and tissue levels (*Figure 2A, B*). Myeloid-specific (LysM) deletion lowered serum OPG without affecting lung tissue, whereas cardiac myocyte-specific (Myl2) deletion decreased OPG levels in serum and lung tissue (*Figure 2A, B*). EC-specific (Cdh5) deletion reduced serum OPG but had no impact on lung levels (*Figure 2C, D*). VSMC-specific (smMHC) deletion caused pronounced reductions in both serum and pulmonary tissue, while FB-specific deletion had only minor effects on serum and no effect on tissue OPG (*Figure 2C, D*).

**Figure 2:**
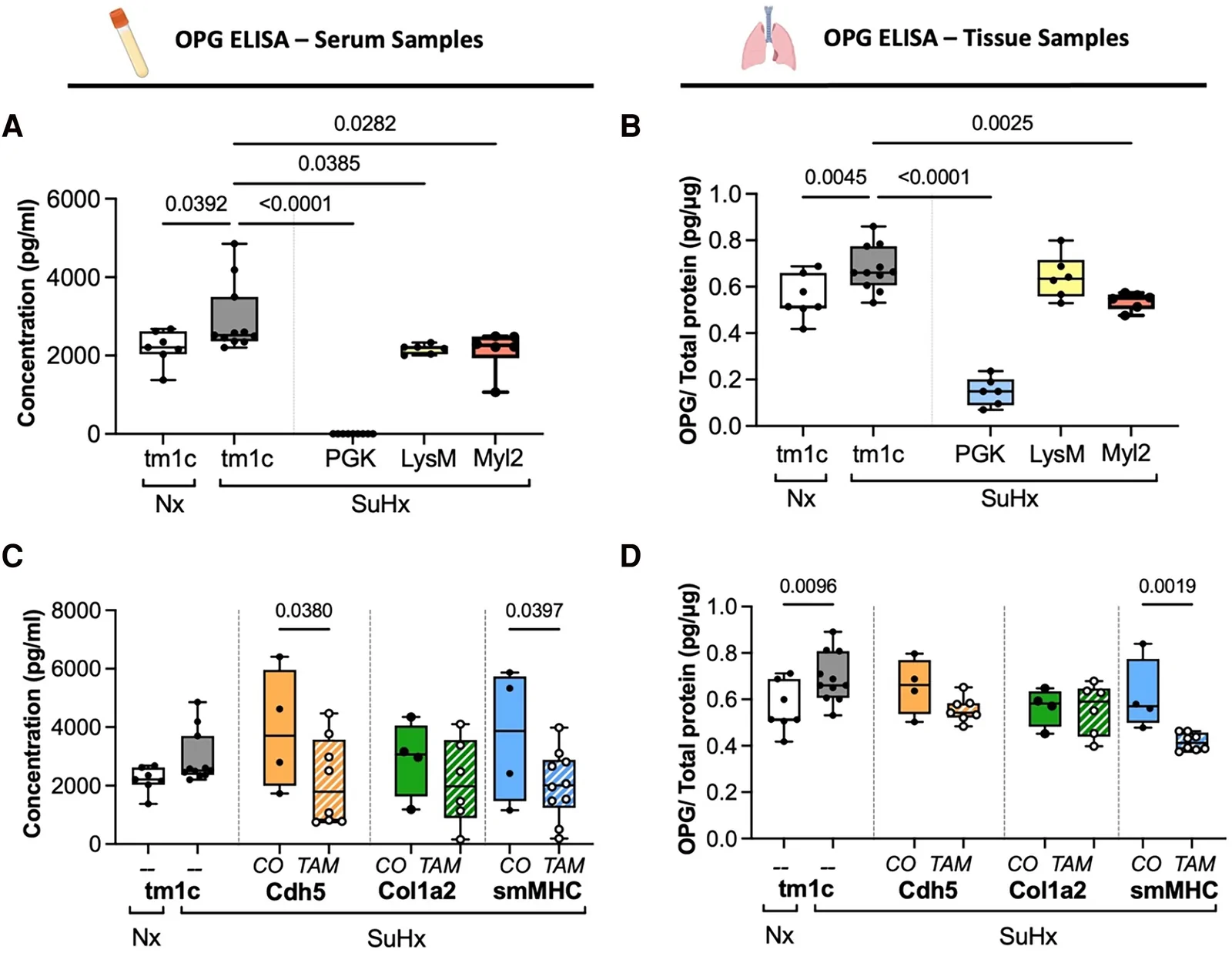
Cell-specific deletion of *Opg* leads to differential OPG levels in serum and lung tissue. (*A*) Circulating OPG levels measured by ELISA in serum collected from control (Nx, *n* = 7), global KO (PGK, *n* = 6–9), myeloid-specific (LysM-Cre, *n* = 6), and CM-specific (Myl2-Cre, *n* = 6) *Opg* deletion mice after 3 weeks of Sugen–hypoxia (SuHx, *n* = 11). (*B*) OPG protein levels in lung tissue homogenates from the same animals, quantified by ELISA and normalized to total protein concentration measured via BCA assay (expressed as pg OPG per μg total protein). (*C* and *D*) Circulating and tissue OPG levels in conditional *Opg* KO mice with cell-specific deletions in ECs [Cdh5-Cre (CO, *n* = 4 and TAM, *n* = 7–8)], smooth muscle cells [smMHC-Cre (CO, *n* = 4, TAM, *n* = 10)], and FBs [*Col1a2*-Cre (CO, *n* = 4, TAM, *n* = 6)]. Data from tm1c control mice in panels (*A*) and (*C*) and panels (*B*) and (*D*) are reused, with plots separated only for representation purposes. Each dot (*n*) represents an individual animal. Data are presented as mean ± SEM and were analysed using one-way ANOVA followed with Fisher’s LSD for multiple comparisons. Statistical significance is indicated by *P*-values.

### 3.2 PGK-cre but not LysM-mediated *Opg* deletion protects against PAH

PGKcre^tg/+^/OPG^fl/fl^ mice (PGK; near-global KO) phenocopied the protective effects of global *Opg* deletion,^25,30^ demonstrating significantly reduced RVSP and estimated pulmonary vascular resistance (ePVR) compared to single transgenic littermate controls (tm1c, SuHx), with no significant changes in left ventricular end-systolic pressure (LVESP) (*Figure 3A–C*; Supplementary material online, *Table S7*). Cardiac output (CO, mL/min) in PGKcre^tg/+^/OPG^fl/fl^ mice was also significantly improved (*Figure 3D*; Supplementary material online, *Table S7*); however, there were no significant changes observed in PA acceleration time (PAAT, ms) and RV hypertrophy (RVH) between both groups compared to SuHx mice (*Figure 3E, F*; Supplementary material online, *Table S7*). IHC analysis of lungs with ABEVG and SMA, to assess pulmonary vascular remodelling revealed that PGKcre^tg/+^/OPG^fl/fl^ mice showed a significant reduction in both the percentage of muscularized arteries, and the degree of muscularization (media/CSA) in small arteries (<50 μm) (*Figure 3G–I*). Considering the involvement of monocytes and macrophages in pulmonary vascular cells during the early onset of disease, and based on our previous findings on bone-marrow chimeric OPG^−/−^ mice suggesting bone-marrow-derived OPG drives PAH development,^25^ we investigated whether myeloid-specific deletion *of Opg* (LysM; LysMcre^tg/+^/OPG^fl/fl^) could protect against PAH progression. However, LysMcre^tg/+^/OPG^fl/fl^ mice did not exhibit any considerable improvements in pulmonary vascular remodelling or haemodynamic parameters (*Figure 3A–D*), suggesting non-myeloid OPG sources drive PAH progression.

**Figure 3:**
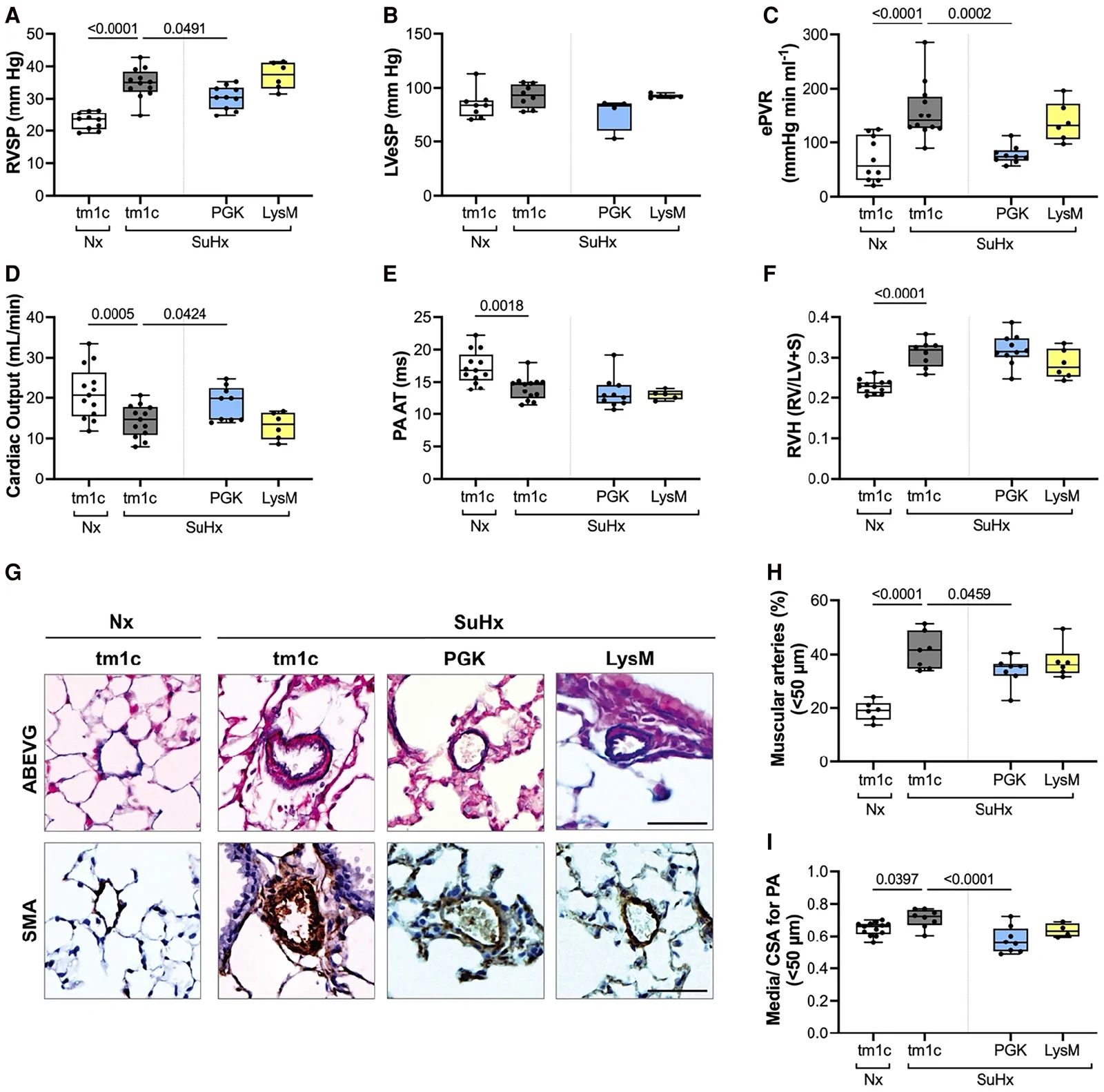
PGKcre^tg/+^/OPG^fl/fl^ mice phenocopied previous results obtained from OPG^−/−^ mice and were protected from developing PAH. Box plots showing (*A*) RVSP (mm Hg), (*B*) LVESP (mm Hg), (*C*) ePVR (mm Hg mL^−1^ min^−1^), (*D*) CO (mL/min), (*E*) PAAT (ms), and (*F*) RVH. Representative images (*G*) of lung sections stained with ABEVG and immunostained for α-smooth muscle actin (α-SMA) following tamoxifen-treatment. Box plots showing the quantification of (*H*) the relative percentage of muscularized pulmonary arteries less than 50 μm, and (*I*) the degree of medial wall thickness as a ratio of total vessel size (media/CSA) in small pulmonary arteries less than 50 μm. [Each dot represents an individual animal (*n*). Nx: *n* = 6–13, SuHx: *n* = 7–13, PGK: *n* = 4–10, LysM-Cre: *n* = 6.] Data are shown as mean ± SEM. *P*-values were calculated with one-way ANOVA followed by Dunnett’s test for multiple comparisons and indicated on the plots.

### 3.3 CM-specific deletion of *Opg* does not prevent PAH progression or cardiac remodelling

Since we also previously reported that OPG is highly expressed in RV of IPAH patients,^25^ we next investigated the role of OPG in cardiac myocytes by generating cardiac myocyte-specific deletion of *Opg* by crossing OPG^fl/fl^ mice with Myl2cre^tg/+^ mice. There were no significant haemodynamic differences between Myl2cre^tg/+^/OPG^fl/fl^ (Myl2) and OPG^fl/fl^ (tm1c) animals following SuHx exposure (*Figure 4A–D*; Supplementary material online, *Table S7*). Analysis of pulmonary vascular remodelling indicated a modest reduction in the percentage of muscularized arteries in Myl2cre^tg/+^/OPG^fl/fl^ mice compared to OPG^fl/fl^ mice after SuHx exposure; however, the degree of remodelling in small arteries remains unchanged between groups (*Figure 4E–G*), and these changes had no impact on haemodynamics. Cardiac function and remodelling were assessed with echocardiography and IF analysis to confirm the changes at the cellular level, respectively. CM CSA (μm^2^) was similar between Myl2cre^tg/+^/OPG^fl/fl^ and OPG^fl/fl^ mice with no considerable changes (*Figure 4H, I*). Additionally, IB4 staining of cardiac capillaries reveal no significant changes in capillary density across experimental groups (*Figure 4J*), suggesting that myocyte-specific deletion of *Opg* has impact on pulmonary vascular and cardiac remodelling during PAH progression induced by SuHx model.

**Figure 4:**
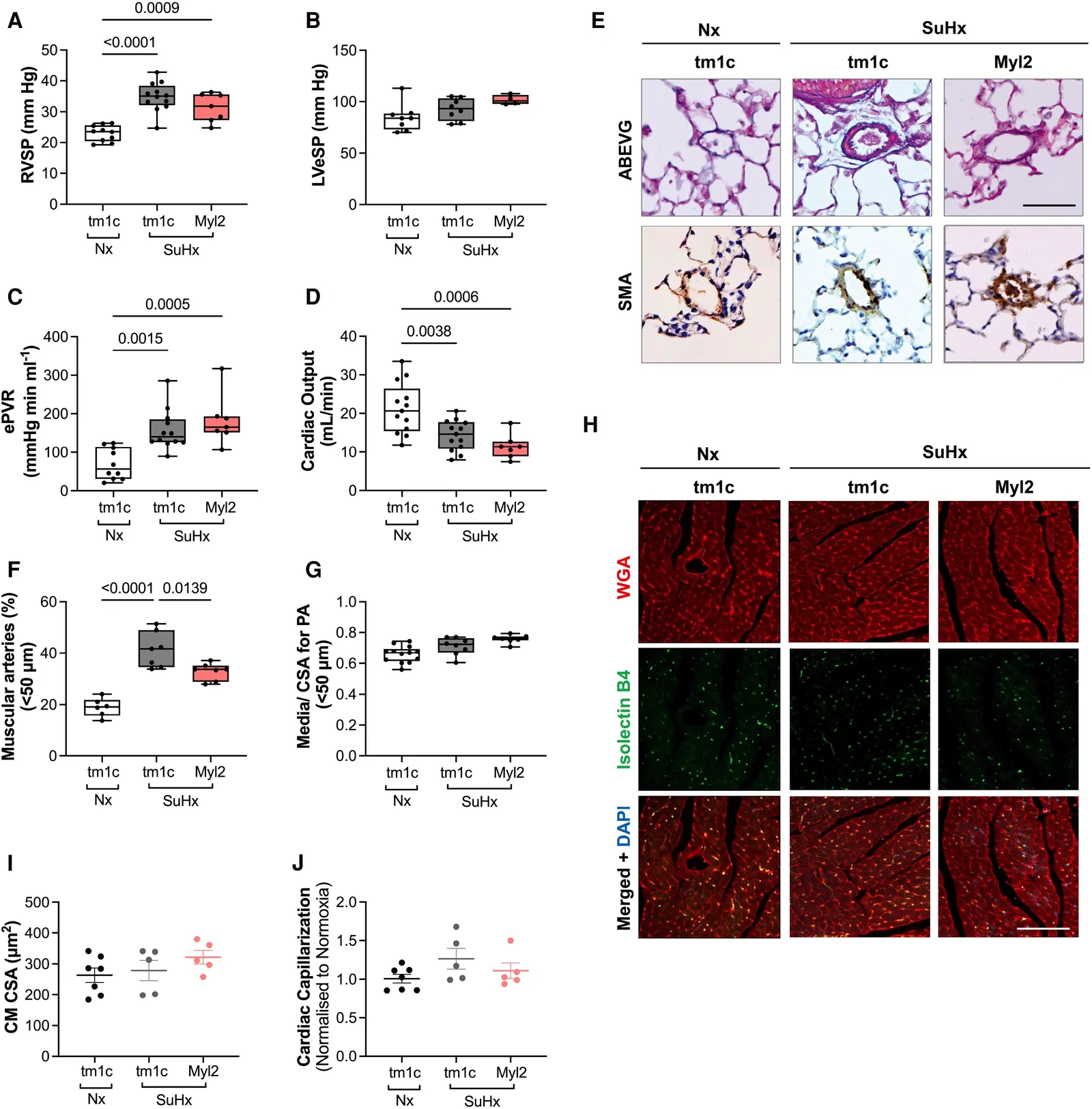
Deletion of OPG in myocardium did not prevent PAH progression or cardiac remodelling. Box plots showing (*A*) RVSP (mm Hg), (*B*) LVESP (mm Hg), (*C*) PAAT (ms), and (*D*) CO (mL/min). Representative images (*E*) of lung sections stained with ABEVG and immunostained for α-smooth muscle actin (α-SMA). Scale bar represents 50 μm. Box plots showing the quantification of (*F*) the relative percentage of muscularized pulmonary arteries less than 50 μm, and (*G*) the degree of medial wall thickness as a ratio of total vessel size (media/CSA) in small pulmonary arteries less than 50 μm. (Panels *A*–*G*: Nx: *n* = 8–10, SuHx: *n* = 8–12, Myl2-Cre: *n* = 4–7.) (*H*) Representative IF images of cardiac tissue samples stained with WGA (WGA coupled with Alexa Fluor 555) and IB4 (IB4 coupled with Alexa Fluor 488). Scale bar represents 100 μm. Each dot represents an individual animal (*n*) showing the quantification of (*I*). CM CSA (μm^2^) and (*J*) myocardial capillary density as normalized to normoxia condition. (Panels *H*–*J*: Nx: *n* = 7, SuHx: *n* = 5, Myl2-Cre: *n* = 5.) Data are shown as mean ± SEM. *P*-values were calculated with one-way ANOVA followed by Tukey’s *post hoc* test for multiple comparisons and indicated on the plots.

### 3.4 Smooth muscle cell-specific deletion of *Opg* predominantly modulates PAH pathogenesis

We next sought to investigate the contribution of EC (Cdh5), VSMC (smMHC), and FB (Col1a2) derived Opg in PAH pathophysiology. There were no significant haemodynamic differences between corn oil and tamoxifen-treated Cdh5cre^tg/+^/OPG^fl/fl^ or Col1a2cre^tg/+^/OPG^fl/fl^ following induction of PAH (*Figure 5A–C*; Supplementary material online, *Table S7*). Mice with VSMC-specific KO of *Opg*, smMHCcre^tg/0^/OPG^fl/fl^ (TAM), demonstrated a significant reduction in RVSP (27.57 ± 4.71 vs. 36.64 ± 1.98 mm Hg, *P* < 0.001) but no difference in LVESP (*Figure 5A, B*; Supplementary material online, *Table S7*). There was a marked reduction in PVR (80.59 ± 33.86 vs. 145 ± 43.82 mm Hg mL^−1^ min^−1^, *P* < 0.05) but no significant effect on CO when compared to corn oil-treated controls following SuHx (*Figure 5C*; Supplementary material online, *Table S7*). EC-specific *Opg* KOs, Cdh5cre^tg/+^/OPG^fl/fl^ mice, exhibited no significant reduction in RVSP, or PVR (*Figure 5A, C*; Supplementary material online, *Table S7*).

**Figure 5:**
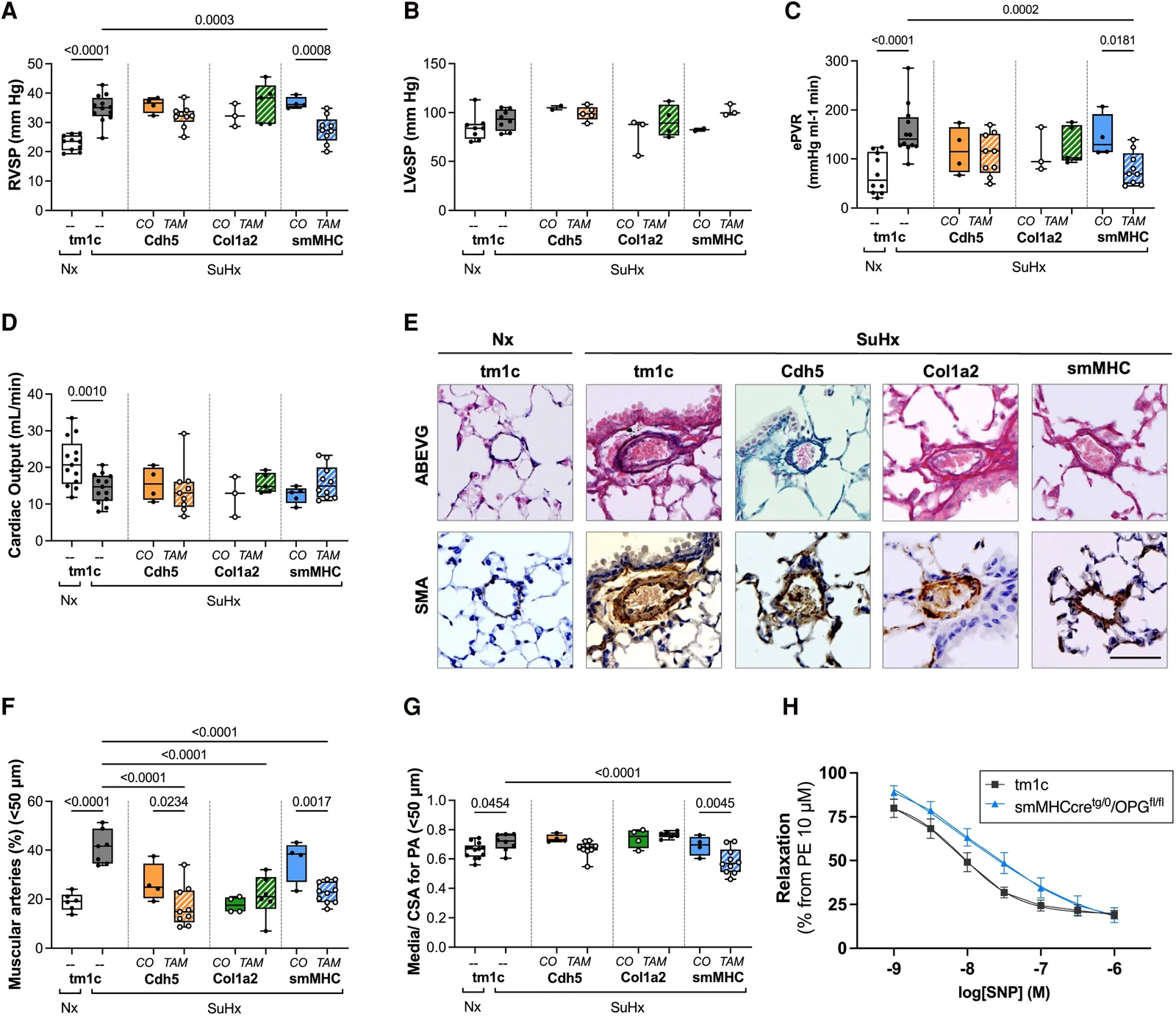
Smooth muscle cell-derived OPG predominantly modulates PAH pathogenesis. Box plots showing (*A*) RVSP (mm Hg), (*B*) LVESP (mm Hg), (*C*) ePVR (mm Hg mL^−1^ min^−1^), and (*D*) CO (mL/min). Representative images (*E*) of lung sections stained with ABEVG and immunostained for α-smooth muscle actin (α-SMA). Scale bar represents 50 μm. Box plots showing the quantification of (*F*) the relative percentage of muscularized pulmonary arteries less than 50 μm, and (*G*) the degree of medial wall thickness as a ratio of total vessel size (media/CSA) in small pulmonary arteries less than 50 μm. Each dot represents an individual animal (*n*), data are shown as mean ± SEM. *P*-values were calculated with one-way ANOVA followed by Fisher’s LSD *post hoc* test and indicated on the plots. (*H*) Vascular reactivity assessed by wire myography. Relaxation–response curve showing contraction force (as % of PE-induced maximum) on the *y*-axis and log-transformed SNP concentration (M) on the *x*-axis. Vessels were pre-contracted with PE, and relaxation in response to SNP was measured to evaluate endothelium-independent vasodilation. [Nx: *n* = 6–13, SuHx: *n* = 6–13, (Cdh5creERT2^tg/+^: CO, *n* = 4, TAM, *n* = 9), (SmMHCcreERT2^tg/0^: CO, *n* = 4, TAM, *n* = 10), (Col1a2creERT2^tg/+^: CO, *n* = 4, TAM, *n* = 5)]. Data shown as mean ± SEM. *P*-values were calculated with one-way ANOVA followed by Fisher’s LSD test.

Assessment of pulmonary vascular remodelling revealed that both EC- and VSMC-specific deletion of *Opg* resulted in a significant reduction in the proportion of muscularized arteries (%) compared to their respective controls (*Figure 5E, F*). In contrast, FB-specific *Opg* KO mice (Col1a2cre^tg/+^/OPG^fl/fl^) were not protected from pulmonary vascular remodelling following SuHx exposure. Notably, only tamoxifen-treated smMHCcre^tg/0^/OPG^fl/fl^ mice exhibited a reduction in vessel wall thickness (media/CSA) (*Figure 5E, G*). Since only VSMC-specific *Opg* deletion led to improvements in both pulmonary vascular remodelling and haemodynamics, we next assessed whether vasoreactivity was altered in those animals compared to corn oil-treated controls. We confirmed that *Opg* deletion did not significantly affect PASMC vasoreactivity (*Figure 5H*).

### 3.5 Smooth muscle cell-derived OPG does not act predominantly via Fas in PAH

We previously identified Fas as a novel receptor for OPG on human PASMCs.^25^ To test whether VSMC-derived OPG signals via the Fas receptor, we generated a mouse line by crossing smMHCcre^tg/0^ mice with Fas^tm1Cgn^ mice to achieve VSMC-specific deletion of *Fas*. These mice were then assessed using the SuHx model to investigate the pathobiological relevance of OPG-Fas signalling in PAH (see Supplementary material online, *Figure S2*).

Haemodynamic analysis revealed that VSMC-specific deletion of *Fas* did not confer any protective effect in the murine SuHx model, with no significant changes observed in haemodynamics or pulmonary vascular remodelling (see Supplementary material online, *Figure S2*). These findings suggest that OPG-Fas binding is not a contributing mechanism by which OPG modulated PASMCs in the mouse SuHx model, indicating the involvement of alternative molecular pathways.

### 3.6 Kinome profiling reveals OPG disruption dysregulates activity of AKT in IPAH-PASMCs

Since only smMHCcre^tg/0^/OPG^fl/fl^ mice exhibited improvements in both pulmonary vasculature remodelling and haemodynamic parameters, we analysed OPG-mediated kinase activity in PASMCs to gain some mechanistic insight. To this end, kinome profiling was performed with primary PASMCs isolated from the lungs of disease-control individuals (donors) and patients with IPAH after lung transplantation. Cells were then stimulated with human recombinant OPG protein, with and without the addition of human anti-OPG monoclonal antibody (see Supplementary material online, *Figure S3A*).

We assessed the activation status of STKs and TKs using the PamGene platform based on the phosphorylation of 144 different substrate peptides for STKs and 196 substrate peptides for TKs (see Supplementary material online, *Figure S3A*). We observed significant differences in phosphorylation levels between PASMCs from donors and IPAH patients, as well as among treatment groups: IgG1-only control (IgG1), OPG-stimulated (OPG + IgG1), and OPG-stimulated with anti-OPG antibody treatment (OPG + mAB:OPG). Heatmaps illustrate the alterations in the phosphorylation levels of TK and STK substrate peptides (*Figure 6A*), which were used to predict the upstream kinase activity of up to 380 protein kinases.

**Figure 6:**
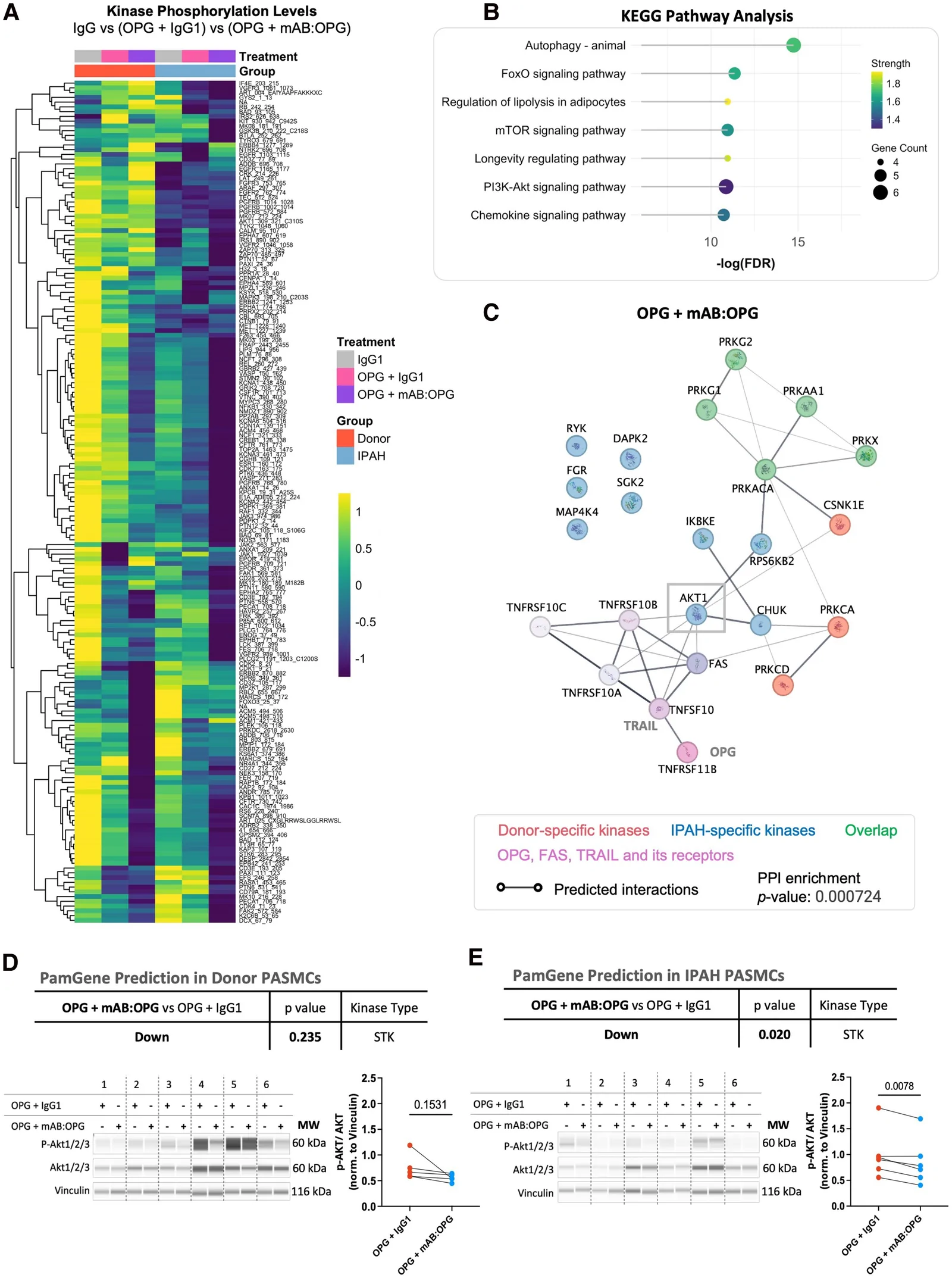
Kinome profiling reveals decreased AKT activity in IPAH HPASMCs following anti-OPG antibody treatment. (*A*) Heatmap representing serine/threonine and tyrosine phosphorylation pattern of the substrates. Mean log-transformed fluorescence signals are shown following OPG stimulation and anti + OPG antibody treatment in PASMCs from disease controls individuals (donor, *n* = 6) and from IPAH patients (*n* = 6). (*B*) Bubble plots showing KEGG pathway enrichment analysis of 17 kinases significantly differentially activated after OPG stimulation followed with anti-OPG treatment (OPG + mAB:OPG). (*C*) PPI map constructed using Cytoscape. Kinases with significantly altered activation specific to donors, IPAH patients, and shared between both were integrated with FAS, OPG, TRAIL, and TRAIL-receptors (TRAIL-R1–3). (*D* and *E*) Validation of AKT1 phosphorylation levels using the Simple Western system. Donor and IPAH samples were run on the same gel and processed in parallel; panels (*D* and *E*) are displayed separately for visual clarity only. Predicted AKT1 activity after OPG stimulation followed anti-OPG treatment (compared to IgG1 controls) was listed in the tables, with quantification was shown as a ratio to total protein. Vinculin was used as a protein loading control. Results are shown as mean ± SEM (*n* = 6) and were compared using a paired *t*-test.

To determine the overall effect of OPG on the kinome, two-group comparisons between OPG-stimulated PASMCs compared to controls (IgG1) were analysed. Across both IPAH- and control-PASMCs OPG stimulation resulted in an overall lower peptide phosphorylation pattern in IPAH-derived compared to control-derived PASMC (*Figure 6A*). We filtered the predicted upstream kinases based on two important parameters: firstly, the mean specificity score is expressed as −log_10_  *P*-value, where *P* < 0.05 refers to the statistical significance for the changes of the phosphorylation of substrate peptide sets between the two selected experimental conditions. Secondly, the predicted differential kinase activity, i.e. median kinase statistic, depicts the overall change of the peptide set that represents the group of substrates for the given kinase (see Supplementary material online, *Figure S3B*, *C*). We subsequently identified 25 kinases (14 in donor and 11 in IPAH-PASMC, Supplementary File  *S1*), which significantly alter their substrate peptide phosphorylation levels following OPG stimulation [e.g Janus Kinase 1 (JAK1), MAPK activated protein kinase 2/3 (MAPKAPK2/3), atrial natriuretic peptide (ANP alpha)], and generated a protein–protein interaction (PPI) network to visualize predicted interactions (see Supplementary material online, *Figures S3B* and *S4A*). Further analysis was conducted to identify signalling pathways affected by the kinases with significantly altered activation levels. Most notably, pathways related to programmed cell death protein-1 and programmed cell death ligand-1 checkpoints, osteoclast differentiation, and platelet and chemokine activation were altered following OPG stimulation (see Supplementary material online, *Figure S4B*). The kinases that were only altered in IPAH patients compared to donor PASMCs were mainly affecting VEGF and MAPK signalling pathways, osteoclast differentiation, and cellular senescence (see Supplementary material online, *Figure S4B*).

Subsequent analysis identified 17 kinases (3 in donor, and 14 in IPAH-derived PASMC, Supplementary File  *S2*) with altered activation following anti-OPG treatment in PASMCs from IPAH patients (e.g. AKT1, death-associated protein kinase 2) and donors (e.g. casein kinase I isoform epsilon, protein kinase C) revealed specific regulation of the FoxO, mTOR, and PI3K–Akt signalling pathways (*Figure 6B*). These changes were accompanied by enrichment in pathways related to autophagy and longevity regulation, both of which are linked to cellular stress resistance and growth (*Figure 6B*).

The 17 kinases with altered activation levels post-anti-OPG treatment were used to construct a PPI network (*Figure 6C*). To explore potential downstream signalling, FAS, OPG, TRAIL, and their receptors were also mapped onto this network. The resulting model suggests that OPG interact with these kinases indirectly through TRAIL and FAS (*Figure 6C*). Among the affected kinases, AKT1 appeared as a central node. Importantly, AKT1 kinase activity was predicted to be significantly reduced in IPAH-PASMCs but not in disease-control samples, which was validated by Simple Western analysis using PASMCs from IPAH patients and disease-control donors (*Figure 6D, E*).

### 3.7 Anti-OPG treatment reduces Akt signalling in rodent models

We previously demonstrated that a human monoclonal anti-OPG antibody treatment reverses PAH in both prophylactic and therapeutic settings in rat SuHx model of PAH.^25^ To validate our findings following the kinome analysis and to further confirm results observed in patient-derived PASMCs, we analysed Akt phosphorylation in lung tissue from SuHx rats (3 weeks hypoxia followed by up to 9 weeks), treated with anti-OPG antibody (KymAb3) (3 mg/kg, three doses) (*Figure 7A*). Western blot analysis revealed that anti-OPG treatment significantly reduced *p*-Akt expression compared to both SuHx and IgG4-treated controls (*Figure 7B, C*), indicating that OPG contributes to Akt pathway activation *in vivo*. Additionally, analysis of p-AKT (Ser473) levels was performed in lung homogenates from VSMC-specific *Opg* KO mice (smMHC-Tam) and corn oil-treated littermate controls (smMHC-CO). Western blotting and quantification (*Figure 7D, E*) showed a reduction in p-AKT levels in smMHC-Tam mice compared with smMHC-CO controls; however, this difference did not reach statistical significance (*P* = 0.0635). Total AKT levels were unchanged between groups.

**Figure 7:**
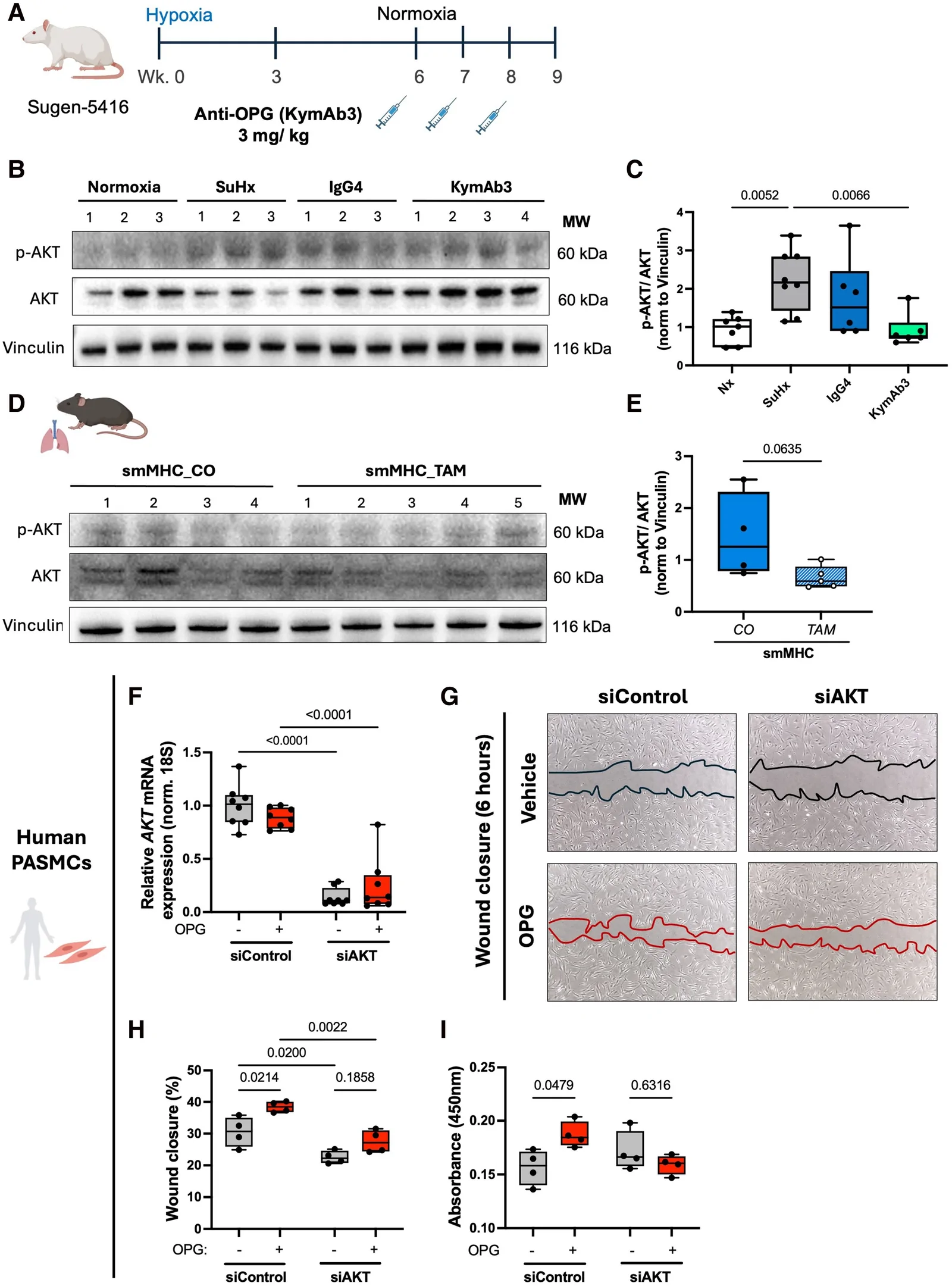
OPG modulates AKT signalling to regulate smooth muscle cell migration and proliferation. (*A*) Schematic of the experimental timeline showing Sugen-5416 and hypoxia (SuHx) exposure in rat, with anti-OPG antibody (KymAb3) treatment administered (3 mg/kg). (*B*) Representative western blot images of phosphorylated Akt (p-Akt), total Akt, and Vinculin in lung tissue from rats under normoxia (*n* = 5), SuHx (*n* = 3), IgG4 control (*n* = 5), and KymAb3 (*n* = 6) treatment conditions. (*C*) Quantification of p-Akt/Akt ratios normalized to Vinculin across experimental groups. Each dot represents an individual animal (*n*); data are presented as mean ± SEM; statistical significance is indicated. (*D*) Representative western blot images of phosphorylated Akt (p-Akt), total Akt, and Vinculin in lung tissue from SmMHCcreERT2^tg/0^ mice (CO *n* = 4, Tamoxifen (Tam) *n* = 5) after SuHx. (*E*) Quantification of p-Akt/Akt ratios normalized to Vinculin across experimental groups. Each dot represents an individual animal (*n*); data are presented as mean ± SEM. (*F*) Relative AKT mRNA expression in siControl- and siAKT-transfected human PASMCs treated with vehicle or OPG. (*G*) Representative images of wound healing assay at 6 h after OPG (or vehicle) stimulation following siControl and siAKT treatments. (*H*) Quantification of wound closure (%). (*I*) Cell proliferation assessed using MTS assay and measured at absorbance (450 nm). For cell culture experiments, each dot represents a biological replicate with three technical replicates. Data shown as mean ± SEM. Statistical significance was determined using one-way ANOVA followed by Tukey’s *post hoc* test.

To examine functional relevance of Akt1 in OPG-induced cell phenotype, we utilized an siRNA approach. Human PASMCs stimulated with OPG recombinant protein following *AKT* siRNA treatment confirmed a significant downregulation of *AKT* in the mRNA levels (*Figure 7F*). Following functional assays indicated that pro-migratory (*Figure 7G* and *H*) and pro-proliferative (*Figure 7I*) effects of OPG stimulation on PASMCs were suppressed after siAKT treatment. Overall, these results indicate that OPG modulation on PASMCs is AKT-dependent.

### 3.8 Tissue OPG levels correlate with RVSP and pulmonary vascular remodelling in VSMC-specific KO mice

To further investigate the relationship between OPG levels and disease severity, we performed correlation analyses in VSMC-specific *Opg* KO mice (*Figure 7F*, Supplementary material online, *Figure S5*). Tissue OPG levels showed strong positive correlations with both RVSP (*R*^2^ = 0.411, Spearman *ρ* = 0.749, *P* = 0.00029) and vascular remodelling (*R*^2^ = 0.373, Spearman *ρ* = 0.682, *P* = 0.00072) (*Figure 7F*). In contrast, serum OPG levels did not correlate significantly with either parameter (see Supplementary material online, *Figure S5*). These findings highlight the importance of local OPG expression in driving pulmonary vascular pathology.

## 4. Discussion

While OPG has previously been implicated in the pathogenesis of PAH, the precise cellular source and the disease specific downstream mechanisms of OPG signalling have remained poorly defined. Here, we utilized a combination of cell-specific conditional *Opg* KO mouse models, primary human (PAH patient-derived) PASMCs, and kinome-wide profiling to systematically understand the role of OPG in PAH progression and pulmonary vascular remodelling. We have shown that among all cell-type-specific conditional KOs generated, only VSMC-specific *Opg* deletion significantly attenuated all markers of disease. In contrast, deletion of *Opg* in myeloid cells, CMs and FBs did not confer any haemodynamic or vascular benefit. Interestingly, while endothelial-specific *Opg* deletion attenuated the number of remodelled pulmonary arteries, it did not reduce the degree of remodelling in affected vessels and was not sufficient to improve haemodynamics. This could be due to the smaller number of ECs in comparison with SMC, and thereby lower local concentrations. Collectively, our findings suggest that VSMC-derived OPG is the predominant contributor to vascular remodelling and haemodynamic deterioration in PAH. These data fit well with our previous work showing high levels of OPG within the media of remodelled pulmonary vascular lesions^23^ and serum,^24^ further emphasizing its utility as a biomarker for PAH development and prognosis.

While our findings provide new insight into the cell-specific roles of OPG in PAH, several limitations should be acknowledged. First, since OPG is naturally secreted, its functional regulation across autocrine and paracrine signalling may not be captured by our model because deleting *Opg* in individual cell populations may be compensated by OPG secreted from other cell types. The use of constitutive cell-specific *Opg* KO models limits our ability to assess the temporal role of OPG during disease progression.

Although we previously proposed OPG and FAS interaction as a main mechanism of action to mediate PASMC proliferation and migration,^25^ our VSMC-specific *Fas* KO model did not replicate the protective phenotype observed with *Opg* deletion. This suggests that FAS might not be the principal mediator of OPG activity in VSMCs within the mouse model studies, and that there are functional differences in Fas signalling or regulation between human and mouse as has been previously reported.^31,32^ Given that VSMC-specific *Fas* deletion was not protective, these data point to alternative upstream mediators of OPG-driven signalling. While our earlier work had identified Fas as the leading candidate receptor, other putative receptors were also described in that study.^25^ Moreover, additional reports show that OPG can interact with integrins (e.g. αvβ3) to activate focal adhesion kinase (FAK)–AKT signalling,^33^ and α5β1 has recently been implicated in vascular remodelling, aligning with the current findings.

Through an unbiased kinome investigation to elucidate downstream signalling networks in primary HPASMCs from IPAH patients, we identified marked alterations in phosphorylation activity across multiple signalling pathways with OPG stimulation followed with antibody blockade. Notably, anti-OPG treatment reduced activity in PI3K–AKT pathway, and autophagy-related pathways that are linked to cell survival and stress resistance. Among these, AKT1 emerged as a central hub for our analysis when we generated a network of OPG, FAS, TRAIL, and TRAIL-receptors (TRAIL-R1, R2, R3, R4) with differentially phosphorylated kinases after anti-OPG treatment following OPG stimulation. Based on kinome profiling, AKT1 kinase activity was significantly suppressed in IPAH-derived PASMCs treated with anti-OPG antibody. Functionally, we demonstrated the dependence of OPG-induced proliferation and migration on AKT. These effects are likely to be more pronounced in hyper-proliferative IPAH-derived PASMC where AKT phosphorylation is increased. These findings are consistent with prior work implicating dysregulated AKT signalling in PAH, in which AKT1 is involved in cell proliferation, survival, and migration^34^ and has been shown to be overactive in PAH.^35^ In a previous study, Jia and colleagues^30^ demonstrated that OPG induce cell proliferation via interacting with integrin α_v_β_3_ leading to downstream FAK and AKT pathway activation in SuHx mouse model of PAH. A recent study from Lemay *et al.*^33^ also studied the role of integrin α_5_β_1_ as a potential therapeutic target for PAH, in which their data suggest that integrin α_5_β_1_ promotes vascular remodelling via Forkhead box protein M1 activation. Although this kinome profiling in PASMCs provided valuable mechanistic insights into OPG-associated signalling, it is important to note that the methodology only captures approximately 70% of known protein kinases. Other signalling mechanisms may also have a role in OPG signalling, limiting our ability to fully identify the full complexity of OPG and its cellular interactions within the pulmonary vasculature. While AKT1 emerged as a key downstream target, broader signalling networks and compensatory pathways remain to be explored.

Our results in the *in vivo* experimental model of SuHx rats treated with monoclonal anti-OPG antibody (KymAb3) further confirms the findings in kinome analysis. This intervention significantly reduced AKT phosphorylation in lung tissue, confirming that OPG contributes to PI3K–AKT pathway activation *in vivo*. Although there was a reduction in pAKT levels in VSMC-specific *Opg* mice, this did not reach statistical significance. This is likely due to exogenous OPG secreted from other cell types, which could still activate AKT within VSMCs.

OPG is primarily defined by its role as a secreted glycoprotein in bone metabolism, where it functions as a decoy receptor for RANKL, thereby inhibiting osteoclastogenesis.^15^ Beyond the skeletal system, OPG has increasingly been recognized for its function in vascular biology, where elevated OPG levels are associated with vascular calcification, arterial stiffness, and increased cardiovascular risk in ageing and chronic inflammatory conditions.^17,18,23,24,36^ Recent studies have identified OPG as a key mediator linking premature VSMC senescence to their phenotypic modulation, in which senescent VSMCs upregulate OPG as a part of the senescence-associated secretory phenotype (SASP),^19^ contributing to the loss of contractile identity and reinforces a synthetic, pro-inflammatory state that impairs their capacity to redifferentiate.^19^ Based on our kinome-wide analysis, blockade of OPG using a monoclonal antibody led to reduced activity of kinases involved in apoptosis, cellular stress, and senescence. These findings align with the growing evidence of positioning OPG as a component of the SASP and suggest a potential role in regulating vascular plasticity.

Additionally, we assessed vascular reactivity using wire myography in pulmonary arteries from wild type and *smMHC-Opg* KO mice. Interestingly, we did not observe significant differences in vasorelaxation responses, suggesting that the protective effects of VSMC-specific *Opg* deletion in PAH are unlikely mediated through changes in contractile function. Instead, our findings support a mechanism whereby VSMC-derived OPG primarily contributes to disease progression via its effects on smooth muscle proliferation, migration, and phenotypic modulation, rather than altering vascular tone directly. Overall, our results indicate that the deletion of *Opg* from VSMCs reduces vascular thickening and restores vascular architecture and may shift VSMCs to a less synthetic or proliferative phenotype. However, it should be noted that our studies were limited to the use of male mice due to the Y-chromosome integration of the *smMHC*-Cre transgene. This approach precludes evaluation of potential sex-specific differences in OPG signalling and vascular remodelling.

Interestingly, correlation analyses performed in VSMC-specific *Opg* KO mice revealed that tissue levels of OPG strongly correlated with both RVSP and pulmonary vascular remodelling, whereas serum levels of OPG did not show any significant association. This suggests that local OPG expression in vasculature, although more challenging to measure, may be a more relevant biomarker of disease severity than circulating levels and further support the notion that VSMC-derived OPG drives disease progression.

Translational perspective This study identifies vascular smooth muscle cell (VSMC)-derived osteoprotegerin (OPG) as a key determinant of pulmonary vascular remodelling and haemodynamic deterioration in pulmonary arterial hypertension (PAH). By defining the cell-specific contribution of OPG, our findings refine current understanding of its pathogenic role and suggest AKT1-dependent signalling as an important mechanism driving PAH pathogenesis in VSMCs. The validation of our findings and attenuation of disease following monoclonal anti-OPG treatment in experimental models reinforces the translational relevance of these findings.

## 5. Conclusion

In summary, our study identifies VSMC-derived OPG as a predominant contributor to pulmonary vascular remodelling and haemodynamic deterioration in experimental PAH. These findings refine prior knowledge derived from global KO models and demonstrate that OPG can promote pathological remodelling via AKT1-dependent signalling rather than Fas in VSMCs. Furthermore, our results support an emerging paradigm in which OPG acts not only as a vascular remodelling mediator but also as a SASP component that links premature senescence to pulmonary vascular pathology. Collectively, these findings provide mechanistic insight and further emphasis to exploring OPG as a translational target in PAH and age-related vascular disease.

## Supplementary Material

cvag197_Supplementary_Data

## Data Availability

The data underlying this article will be shared on reasonable request to the corresponding author.
