# Supplementary material for: Vascular smooth muscle cell-derived osteoprotegerin drives pulmonary arterial hypertension via AKT activation and is attenuated by monoclonal antibody therapy
Source: Cardiovasc Res. 2026 Sep 7;122(15):2189–203. doi: 10.1093/cvr/cvag197 (PMC13742051; doi:10.1093/cvr/cvag197)
Supplement: Supplementary file 1 [file cvag197_supplementary_data.zip › CVR-2025-1756_Supplementary Material_R2_v2_Clean.docx]

**Vascular Smooth Muscle Cell–Derived Osteoprotegerin drives pulmonary arterial hypertension via AKT activation and is attenuated by monoclonal antibody therapy**

**Keles et al**

**Supplementary Material**

1. Supplementary Methods and Tables
2. Supplementary Figures

**1. Supplementary Methods and Tables**

### 1.1. **Genomic DNA Isolation and Genotyping**

Transgenic mouse colonies were generated by crossing the indicated mouse genotypes in the **Supplementary** **Table 1.** Mouse colonies were consequently genotyped for floxed alleles by using the primers given in **Supplementary Table 2**. Genomic DNA was extracted from mouse ear punch tissue using the REDExtract-N-Amp Tissue PCR Kit (Sigma Aldrich, XNAT). Briefly, tissue samples were lysed in Extraction Solution (Sigma Aldrich, E7526) and incubated at room temperature for 10 minutes, followed by heat block incubation at 95°C for 3 minutes. The lysate was then mixed with Neutralization Solution B (Sigma Aldrich, N3910) and vortexed. The extracted DNA was used for PCR amplification according to the manufacturer’s protocol. The amplified DNA was subsequently analysed by agarose gel electrophoresis for genotyping. Primers used for detecting transgenic animals and internal controls are listed in **Supplementary** **Table 2**.

**Supplementary Table 1: List of experimental transgenic mouse lines and description.**

| Full Strain Name | Name used in the study | Description |
| --- | --- | --- |
| C57BL/6NTac-*Tnfrsf11b^tm3877Tac^* | OPG^fl/fl^ |  |
| B6.C-Tg(Pgk1-cre)1Lni/CrsJ | PGKcre | Cre recombinase under the control of the phosphoglycerate kinase 1 (PGK1) promoter in all tissues |
| B6.129P2-Lyz2^tm1(cre)Ifo^/J | LysMcre | Myeloid-cell specific expression of constitutive Cre recombinase |
| CBB10-Tg(myl2-cre)1118Tmhn/H | Myl2cre | Myocardial-specific expression of Cre recombinase (tamoxifen-inducible) |
|  | Cdh5creERT2 | Endothelial cell-specific expression of Cre recombinase (tamoxifen-inducible) |
| B6.FVB-Tg(Myh11-cre/ERT2)1Soff/J | smMHCcreERT2 | VSMC-specific expression of Cre recombinase (tamoxifen-inducible)  *Tg inserted on Y-chromosome |
| B6.Cg-Tg(Col1a2-cre/ERT,-ALPP)7Cpd/J | Col1a2creERT2 | Fibroblast-specific expression of Cre recombinase (tamoxifen-inducible) |
| B6.Cg-*Fas^tm1.1(ALPP)Chnd^/J* | Fas^fl/fl^ | Exons 2-9 of the mouse *Fas* gene are flanked by loxP sites followed by Cre-mediated recombination |

**Supplementary** **Table 2: List of primers used for genotyping transgenic colonies.**

| Target | Primer type | Sequence (5’ to 3’) |
| --- | --- | --- |
| OPG^fl/fl^ | Detects the flanked allele - LoxP prox_F | GTAGAGCTGTATCTTGGGTCCC |
|  | Detects the flanked allele - LoxP dist_F | CAACTCACTGCTCTTTACACAGC |
|  | Detects the flanked allele - LoxP dist_R | TTAGCCTCAACGGTGTGACC |
| Pgk1-cre | Transgene forward | TTCTCCTCTTCCTCATCTCCG |
|  | Transgene reverse | GCAAACGGACAGAAGCATTT |
|  | Internal control forward | CTAGGCCACAGAATTGAAAGATCT |
|  | Internal control reverse | GTAGGTGGAAATTCTAGCATCATCC |
| LysM-cre | Transgene forward | CCCAGAAATGCCAGATTACG |
|  | Mutant/ control reverse | CTTGGGCTGCCAGAATTTCTC |
|  | WT control forward | TTACAGTCGGCCAGGCTGAC |
| Myl2-cre | Transgene forward | TGGAATGGACCTAGGGGGAG |
|  | Transgene reverse | AGGTTCTGCGGGAAACCATT |
| smMHC-creERT2 | Transgene forward | TGACCCCATCTCTTCACTCC |
|  | Transgene reverse | AGTCCCTCACATCCTCAGGTT |
|  | Internal control forward | CAGCCAACTTTACGCCTAGC |
|  | Internal control reverse | TCTCAAGATGGACCTAATACGG |
| Cdh5-creERT2 | Transgene forward | TCCTGATGGTGCCTATCCTC |
|  | Transgene reverse | CCTGTTTTGCACGTTCACCG |
| Col1a2-creERT2 | Transgene forward | ATTGGTTTGAACCAGGCAGT |
|  | Transgene reverse | CGGTTATTCAACTTGCACCA |
| Fas^fl/fl^ | Transgene forward | GCAAGAAGGAAGCACAGACA |
|  | Transgene reverse | CAGGCTCCGACTCATCATCT |

### 1.2. **Transthoracic Echocardiography**

Echocardiography was recorded with a linear 30- to 40-MHz transducer (MX-550D) and the Vevo 3100 system (VisualSonics, Toronto, Canada). Animals were placed dorsally on a heated table to maintain the body temperature during the procedure, while ECG and respiration rate were continuously recorded. Anaesthesia was induced and maintained using isoflurane (maintenance at 0.5-1.0% after induction with 2% isoflurane) to sustain heart rates at 450–500 bpm in mice.

Animals were depilated, and pre-heated ultrasound gel (Aquasonics 100 Gel, Parker Labs Inc., Fairfield, NJ) was applied for the procedure. B-mode and M-mode tracings were recorded in both parasternal long-axis (PSLAX) and short-axis (PSAX) views at the level of papillary muscle and pulmonary artery, respectively. The change in left ventricular diameter length from end-diastole to end-systole (LVID) was used to assess contractility and to calculate LV ejection fraction and fractional shortening (LV-EF and LV-FS measured using the PSLAX mode. The right ventricle was visualised in the right parasternal long axis, where right ventricular free wall thickness (RVFWT) and right ventricular internal diameter (RVID) were measured at end-diastole phase to assess RV microanatomy. Pulsed-waved (PW) Doppler measurements were utilized to determine the peak flow in the PA, and PA acceleration time (PA AT) and ejection time (PA ET) were measured from the PW diagram. Data were analysed with the Vevo Lab software (VisualSonics, v5.5.0).

### 1.3. **Haemodynamic Measurement of Right and Left-ventricular Pressure**

Left and right ventricular catheterisation was performed under isoflurane-induced anaesthesia (maintenance at 1.5% after induction with 3% isoflurane) by using a closed chest method via the right internal carotid artery and right external jugular vein. Pressure-volume measurements were recorded using PVR-1045 1F (LV) and PVR-1030 1F (RV) catheters (Millar Inc.), connected to a Millar MPVS Ultra and PowerLab 8/30 data acquisition system (AD Instruments Ltd, Oxford, UK). Data were recorded with LabChart v7 software (AD Instruments Ltd) and analysed using PVAN v2.3 (Millar, Houston, TX, USA).

Estimated pulmonary vascular resistance (ePVR) was calculated using the following equation: Estimated mean pulmonary artery pressure (EmPAP) — left ventricular end-diastolic pressure (LVEDP)/cardiac index). EmPAP was derived by substituting systolic PAP for RVSP, in the following equation [EmPAP = (0.61 x RVSP) + 2 mmHg].^20^ Following haemodynamic measurements, animals were maintained under deep anaesthesia with isoflurane inhalation (4%) and euthanasia was performed by exsanguination with cardiac puncture followed by cervical dislocation in accordance with institutional and national ethical guidelines for animal care and use. Tissues were then harvested for the analyses described below.

### 1.4. **Blood and Organ Collection**

Following right and left ventricular catheterisation, animals were euthanized under anaesthesia to collect blood via intra-cardiac puncture. The right ventricle (RV) was injected with 2 ml of PBS to flush the remaining blood from the pulmonary vasculature. The heart, lungs, and trachea were then removed en bloc. The right lung was ligated and removed, whilst the left lung was perfusion-fixed via the trachea with 10% (v/v) formalin-buffered saline by inflation to 20 cm H₂O. The left lung and heart were fixed for 24 hours at 4°C in 10% (v/v) neutral buffered formalin. After fixation, the heart and lungs were processed into paraffin blocks for sectioning.

## 1.5. **Histological and Immunohistochemical Staining**

Dual chromogenic IHC staining was performed combining the HRP based DAB substrate (ImmPACT® DAB Substrate Kit, HRP, Vector Laboratories, USA) and an alkaline phosphatase (AP) based substrate (ImmPACT® Vector® Red Substrate Kit, AP, Vector Laboratories, USA). Both kits were used as per manufacturer’s recommendations and optimized to the primary antibody. For mouse tissue-based staining a mouse-on-mouse (M.O.M.® blocking reagent, Vector Laboratories, USA) blocking step was included. Further, an endogenous peroxidases blocking step (BLOXALL®, Vector Laboratories, USA) was added. Primary antibodies were incubated and developed sequentially using appropriate HRP- or AP- linked secondary antibodies. Blue Haematoxylin was used as nuclear counterstain (from Haematoxylin and Eosin Kit, Vector Laboratories, USA).

Haematoxylin and Eosin staining was performed using a ready-to-use kit (Haematoxylin and Eosin Kit, Vector Laboratories, USA) as per manufacturer’s recommendations. IHC was performed for α-SMA using HRP based DAB substrate (ImmPACT® DAB Substrate Kit, HRP, Vector Laboratories, USA) with the appropriate HRP linked secondary antibody. Blue Haematoxylin was used as nuclear counterstain (from Haematoxylin and Eosin Kit, Vector Laboratories, USA).

**Supplementary** **Table 3: List of antibodies used in immunohistochemistry (IHC) and immunofluorescence (IF) staining.**

| **Target** | **Company/ Catalogue Number** | **Application** |
| --- | --- | --- |
| Isolectin B4 (IB4) | Vector Laboratories, FL-1201 | IF (1:50) |
| ProLong™ Gold Antifade Mountant with DNA Stain DAPI | Invitrogen, P36935 | IF |
| Wheat Germ Agglutinin (WGA) | Invitrogen, W21405 | IF (1:100) |
| α-Smooth Muscle Actin (α-SMA) | Sigma Aldrich, A5228 | IHC (1:200) |
| Osteoprotegerin (OPG) | Abcam, ab73400 | IHC (1:200) |
| CD31/PECAM-1 | bio-techne, AF3628 | IHC (1:100) |
| ImmPACT® DAB Substrate Kit, HRP | Vector Laboratories, SK-4105 |  |
| ImmPRESS® HRP Horse Anti-Mouse IgG | Vector Laboratories, MP-7402 |  |
| Anti-Rabbit IgG (whole molecule)–Peroxidase antibody produced in goat | Sigma Aldrich, A6154 | IHC (1:500) |
| Rabbit Anti-Goat IgG H&L (HRP) | Abcam, ab6741 | IHC (1:500) |
| Hematoxylin Counterstain from H&E Kit | Vector Laboratories, H-3502 |  |

### 1.6. **Quantification of Pulmonary Vascular Remodelling**

Pulmonary vascular remodelling was evaluated by measuring the extend of muscularization and the percentage of affected pulmonary arteries and arterioles. For each lung pulmonary arteries were classified as either muscularized (having crescent or complete rings of muscle) or non-muscularized (lack of apparent muscle) based on ABEVG stained sections. Vessels with external diameter smaller than 50 µm were quantified. The proportion of muscularised vessels in each sub-group was expressed as a percentage of the total number of vessels. Additionally, the degree of muscularisation for each group was also quantified as the medial area of the vessel divided by the total vessel cross-sectional area (media/CSA). One hundred vessels were quantified for each analysis, and the results were averaged to represent one biological replicate.

### 1.7. **Quantification of Cardiac Remodelling**

Cardiac remodelling was evaluated by measuring the cardiomyocyte cross-sectional area (CSA) in WGA-stained sections to assess cellular hypertrophy. For each analysis, two hundred cardiomyocytes from four distinct heart regions were quantified, and the results were averaged to represent one biological replicate. Cardiac capillarization was assessed by counting IB4+ cells in four standardized regions of equal size, with results normalized to the normoxia condition. Additionally, if the heart was not sectioned for histological analysis, right ventricular hypertrophy (RVH) was assessed by calculating the ratio of the right ventricular free wall weight (RVFW) to the combined weight of the left ventricle and septum (LV + S) using the formula: RVH = RVFW / (LV + S).

1.8. **Cell culture**

***Validation of Kinome Study:*** Human PASMCs were cultured in smooth muscle growth medium-2 (SmGM-2, CC-3182, Lonza) supplemented with fetal bovine serum (FBS), human fibroblast growth factor-B (hFGF-B), human epidermal growth factor (hEGF), insulin, and gentamicin/amphotericin-B, at 37 °C in a humidified incubator with 5% CO_2_. All experiments were performed using cells between passage 4 to 6.

Before stimulation, cells were synchronized for 48 hours with smooth muscle basal medium (SmBM, CC-3181, Lonza) with 0.2% FBS and no additional growth factors. Cells were then stimulated with 50 ng/ml recombinant human OPG protein (185-OS-025/CF, Bio-Techne Ltd, R&D Systems) supplemented in SmBM, either alone or in combination with human anti-OPG antibody (MAB805, Bio-Techne Ltd, R&D Systems), or recombinant human IgG1 Fc protein (CF, 110-HG**,** Bio-Techne Ltd, R&D Systems) as a control for 30 minutes.

**Supplementary Table 4: Demographic characteristics of patient-derived pulmonary artery smooth muscle cells (PASMCs) isolated from disease control (Donor) and idiopathic pulmonary arterial hypertension (IPAH) patients.**

| Condition/ Sample ID | Date of Sampling | Age [years] | Sex | Pathology report |
| --- | --- | --- | --- | --- |
| Donor_1 | 08.11.2021 | 51 | M | Emphysema grade 4-5 (Thurlbeck), mild chronic bronchitis, condensate macrophages, pulmonary vessels unaffected |
| Donor_2* | 05.08.2015 | 34 | F | Mild chronic bronchitis, severe emphysema grade 7-9 (Thurlbeck), focal subpleural interstitial fibrosis vasculopathy grade 1 (Heath/Edwards) |
| Donor_3 | 09.04.2019 | 59 | F | COD: Intracranial bleeding, non-smoker |
| Donor_4* | 28.02.2020 | 43 | M | emphysema grade 5-6 (Thurlbeck), mild chron. bronchitis with peribronchial mixed-dust anthracosis, vasculopathy grade 1 (Heath/Edwards) |
| Donor_5* | 14.11.2020 | 54 | F | emphysema grade 6-7 (Thurlbeck), areas with dystelectasis and alveolar oedema, focal chronic interstitial alveolitis, condensate macrophages (> nicotine abuses) |
| Donor_6* | 15.12.2020 | 59 | F | minimal chronic bronchitis, emphysema grade 6-7 (Thurlbeck), condensate macrophages, subpleural interstitial scars |
| IPAH_1 | 27.22.2012 | 39 | F | sclerosis of pulmonary arteries, intima proliferation of resistance vessels, plexiform lesions, vasculopathy grade 3-4 (Heath/Edwards), minimal chronic bronchitis, emphysema grade 3-4 (Thurlbeck) |
| IPAH_2 | 23.04.2015 | 41 | M | PAH, sclerosis of pulmonary arteries, media hypertrophy, intimal proliferation with stenosis, plexiform lesions, vasculopathy grade 3-4 (Heath/Edwards), moderate emphysema grade 3-4 (Thurlbeck) |
| IPAH_3 | 24.10.2017 | 50 | M | chronic PAH, severe sclerosis of pulmonary arteries, plexiform lesions, focal bleedings into lung parenchyma, mild chronic bronchitis |
| IPAH_4 | 23.11.2017 | 17 | F | moderate - severe pulmonary hypertension, focal plexiform lesions, mild derangement of lung architecture (subpleural, para-septal) |
| IPAH_5 | 23.10.2018 | 17 | F | severe pulmonary hypertension, plexiform lesions, vasculopathy grade 5 (Heath/Edwards), perivascular fibrosis |
| IPAH_6 | 22.11.2018 | 50 | F | sclerosis of pulmonary arteries, intima proliferation with stenosis of resistance vessels, plexiform lesions, vasculopathy grade 3-4 (Heath/Edwards), mild emphysema grade 4 (Thurlbeck), focal interstitial fibrosis and leiomyomatas-like proliferation |

Date: Date of lung transplantation or reception in the biobank

*: Subarachnoid haemorrhage as cause of death

***Wound-healing Assay:*** Cell migration was assessed using a wound healing assay in human PASMCs (Lonza, CC-2581, Donor IDs: 22TL073026 and 22TL221791). Cells were seeded in 6-well plates and cultured in smooth muscle basal medium supplemented with growth factors and 5% FCS until reaching 70–80% confluence. Cells were then transfected with control or AKT1 (ThermoFisher Scientific SilenceSelect siRNA, Assay ID: s659) siRNA in OPTI-MEM for 4 hours. Following transfection, cells were cultured in medium containing 0.2% FCS to synchronize them prior to OPG stimulation (50 ng/ml).

To inhibit cell proliferation, cells were pre-treated with Mitomycin C (10 µg/mL) for 2 hours prior to scratch generation. Cells were then washed with PBS to remove residual mitomycin C. A linear scratch was generated across the cell monolayer using a sterile 200 µL pipette tip. Detached cells were removed by gently washing twice with PBS, and cells were maintained in serum-reduced medium. Where indicated, cells were stimulated with OPG or vehicle control immediately following scratch formation.

Phase-contrast images were acquired immediately after scratch generation (0 h) and at subsequent time points. Wound closure was quantified by measuring the percentage of wound closure using ImageJ.

***Cell proliferation assay:*** Cell proliferation was assessed using the CellTiter 96 AQueous One Solution Cell Proliferation Assay (Promega, G3580) according to the manufacturer’s instructions. Human PASMCs were seeded in 96-well plates and cultured in smooth muscle basal medium supplemented with growth factors and 5% fetal calf serum (FCS). Cells were transfected with control or AKT1 siRNA in OPTI-MEM for 4 h, followed by incubation in medium containing 0.2% FCS to synchronize the cells prior to stimulation.

Cells were then treated with vehicle control, OPG (50 ng/ml) or with PDGF (20 ng/ml) as a positive control. At the end of the treatment period, 20 µL of the MTS reagent was added to each well containing 100 µL of culture medium and incubated for 3 hours at 37 °C. Absorbance was measured at 450 nm using a microplate reader. Cell proliferation was calculated relative to control conditions after background subtraction*.*

## **Simple-Western**

Jess Simple Western system (ProteinSimple, San Jose CA, USA) is an automated capillary-based size separation immunoassay to detect proteins of interest in low µg amounts. According to the manufacturer's instructions, 2.4 µL of protein lysate was mixed with 0.6 µL of 5x fluorescence master mix which is provided as part of the standard reagent pack. Samples were denatured for 5 minutes at 95°C and stored on ice. Samples as well as all further components, i.e. antibodies for immunodetection, wash buffer, BSA blocking solution, Replex buffer for re-probing, were loaded onto the plate which was afterwards centrifuged for 5 minutes at 1,000 *g*. The plate and the cartridge containing the capillaries for proteins with a molecular weight of 12 to 230 kDa were placed in the Jess device. A fully automated separation followed by an immunodetection based on chemiluminescence was performed within 5 hours. Light emission was detected using a CCD camera. Results, i.e. protein expression reflected by band intensities, are displayed in traditional lane view as well as in electropherograms. The latter ones allow for densitometric quantification by defining the area under the curve for the respective protein of interest using the Compass software (version 4.1.0, Protein Simple). Normalized ratios of expression are calculated as indicated in the figure legends. Antibodies used in Simple Western methodology are listed in **Supplementary Table 5.**

**Supplementary Table 5: Antibodies used for Simple Western.**

| Target | Company | Article No. | Source | Dilution |
| --- | --- | --- | --- | --- |
| Phospho-Akt (Ser473) | Cell signaling | #9271 | Rabbit | 1/10 |
| Akt Antibody | Cell signaling | #9272 | Rabbit | 1/50 |
| Vinculin | Sigma-Aldrich | #V9131 | Mouse | 1/1250.000 |

## 1.1. **Western Blotting**

Lung tissue samples were obtained from our previously published study investigating the therapeutic effects of a monoclonal anti-OPG antibody (KymAb3), where the detailed treatment protocols, including dosage and administration schedule are available in that publication.^25^

Tissue lysates were prepared and mixed with sample buffer (NuPAGE LDS Sample Buffer, Invitrogen, NP0007) and reducing agent (NuPAGE Sample Reducing Agent, Invitrogen, NP004). Equal amounts of total protein (15–20 µg) were separated on 4–12% precast polyacrylamide gels (NuPAGE Bis-Tris Mini Gels, Invitrogen, NP0321). Proteins were transferred to membranes, which were blocked in 5% (w/v) BSA in TBST (Pierce Concentrated Buffer Stocks, Thermo Fisher Scientific, 28360) and incubated with primary antibodies overnight at 4 °C, followed by a 1-hour incubation at room temperature with the appropriate secondary antibodies **(Supplementary Table 6)**.

**Supplementary Table 6: List of antibodies used in Western blotting.**

| Target | Company/ Catalogue Number | Application |
| --- | --- | --- |
| Phospho-Akt1 (Ser463) – D7F10 XP | Cell Signaling, 9018S | 1:1000 |
| Akt1 (D9R8K) | Cell Signaling, 75692S | 1:1000 |
| Vinculin | Cell Signaling, 13901S | 1:5000 |
| b-actin | Proteintech, 66009-1 | 1:5000 |
| Anti-Mouse Secondary Antibody | Cytiva, NA931 | 1:2000 |
| Anti-Rabbit Secondary Antibody | Sigma, A6154 | 1:5000 |

**Supplementary Table 7:** Summary of haemodynamic parameters across all mouse lines and treatment conditions used in the study. Data are shown as mean ± SD.

| Genotype | RVSP (mmHg) | mPAP (mmHg) | LVeSP (mmHg) | LVeDP (mmHg) | CO (mL/min) |
| --- | --- | --- | --- | --- | --- |
| tm1c-Nx | 23.05 ± 2.62 | 16.06 ± 1.60 | 84.71 ± 13.35 | 6.32 ± 2.41 | 21.28 ± 6.54 |
| tm1c-SuHx | 34.83 ± 4.67 | 23.59 ± 2.33 | 92.66 ± 10.54 | 8.26 ± 2.98 | 14.32 ± 3.93 |
| PGK-SuHx | 30.45 ± 3.60 | 21.02 ± 1.73 | 76.42 ± 15.97 | 6.93 ± 3.56 | 18.61 ± 4.26 |
| LysM-SuHx | 37.11 ± 4.15 | 23.82 ± 2.35 | 92.48 ± 1.86 | 8.30 ± 2.11 | 13.10 ± 3.32 |
| Myl2-SuHx | 31.37 ± 4.59 | 21.13 ± 2.80 | 100.80 ± 3.13 | 6.26 ± 0.66 | 11.40 ± 3.20 |
| Cdh5-CO-SuHx | 35.98 ± 2.63 | 23.95 ± 1.60 | 104.90 ± 2.83 | 7.47 ± 3.08 | 15.50 ± 4.60 |
| Cdh5-Tam-SuHx | 32.13 ± 3.75 | 21.60 ± 2.29 | 99.17 ± 7.22 | 9.56 ± 3.08 | 13.90 ± 6.59 |
| smMHC-CO-SuHx | 36.64 ± 1.98 | 23.93 ± 1.55 | 82.57 ± 2.01 | 5.72 ± 0.73 | 12.45 ± 2.25 |
| smMHC-Tam-SuHx | 27.57 ± 4.71 | 18.35 ± 3.10 | 102.10 ± 5.86 | 6.88 ± 1.15 | 15.92 ± 4.50 |
| Col1a2-CO-SuHx | 32.45 ± 3.91 | 21.09 ± 1.41 | 77.83 ± 19.24 | 8.84 ± 1.88 | 12.27 ± 5.51 |
| Col1a2-Tam-SuHx | 36.55 ± 6.94 | 24.29 ± 4.24 | 91.35 ± 16.47 | 4.60 ± 2.80 | 15.69 ± 2.70 |

**2. Supplementary Figures**

**Supplementary Figure 1: Tamoxifen‑induced lineage‑specific deletion of OPG in SuHx‑exposed mouse lungs.**

Representative immunostaining of lung sections from SuHx mice receiving corn oil (top row) or tamoxifen (OPG knockout induction; bottom row). OPG (red) is shown together with markers labelling: CD31 for endothelial cells (left, brown), Col1a1 for fibroblast‑associated collagen‑producing cells (middle, brown), and αSMA for smooth muscle cells (right, brown). Scale bar represents 50 mm.

**Supplementary Figure 2: Smooth muscle cell-derived OPG does not act predominantly via Fas in PAH**

Box plots showing **a.** right ventricular systolic pressure (RVSP, mm Hg), **b.** left ventricular end-systolic pressure (LVESP, mm Hg), **c.** estimated pulmonary vascular resistance (ePVR, mmHg ml^-1^ min)**, d.** cardiac output (CO, ml/min), **e.** pulmonary artery acceleration time (PA AT, ms), **f.** right ventricular hypertrophy (RVH). Representative images **g.** of lung sections stained with Alcian Blue Elastic van Gieson (ABEVG) and immunostaining for α-smooth muscle actin (α-SMA). Scale bar represents 50 µm. Box plots showing the quantification of **h.** the relative percentage of muscularised pulmonary arteries less than 50 µm, and **i.** the degree of medial wall thickness as a ratio of total vessel size (Media/CSA) in small pulmonary arteries pulmonary arteries less than 50 µm. Data are shown as mean ± SEM. *P*-values were calculated with one-way ANOVA followed by Dunnett’s test for multiple comparisons and indicated on the plots.

**Supplementary Figure 3: Peptide-based kinome profiling identified several protein kinases regulated upon activation by OPG stimulation and following anti-OPG antibody treatment.**

**a.** Experimental scheme showing the application of kinome-wide analysis using the PamStation platform. Results from the upstream kinase prediction based on differential peptide phosphorylation signatures are shown in **b.** IgG vs OPG+IgG1 and **c.** OPG+IgG1 vs. OPG+mAB:OPG treated human PASMCS. Only kinases that met the criteria of significance in the change of phosphorylation of the sets of substrate peptides for the given kinase between the two experimental conditions are depicted in bubble plots. The x-axis of the plot represents the mean specificity score while the size of the bubble represents the mean kinase statistic and the colour indicating the p-value.

**Supplementary Figure 4: OPG stimulation of PASMCs results activation of kinases related to cellular senescence and osteoclast differentiation.**

**a.** Protein-protein interaction (PPI) map constructed using Cytoscape. Kinases with significantly altered activation are shown specific to donors (red), IPAH patients (blue) and shared between both (green). **b.** Bubble plot showing KEGG pathway enrichment analysis of 25 kinases significantly differentially activated following OPG stimulation (OPG+IgG) compared to IgG control condition. The x-axis of the plot represents -log(FDR), the size of the bubble represents the number of the genes, and the colour indicates the strength (normalised enrichment score).

**Supplementary Figure 5: Tissue OPG levels are correlated with haemodynamics and pulmonary vascular remodelling parameters.**

Correlation analysis between serum (a) and tissue (b) OPG levels with right ventricular systolic pressure (RVSP) and vascular remodelling (Medial to cross-sectional area ratio). Data points are color-coded by treatment group (Corn-oil (CO): pink and Tamoxifen (Tmx): blue), with linear regression lines (shaded region indicates confidence interval (95% CI) and Spearman correlation coefficients (r) and statistics (p-value) are shown.

**Supplementary Figure 6: Unedited Simple Western images corresponding to Figure 6d-e.**
Uncropped and unprocessed Simple Western images showing detection of phosphorylated AKT1/2/3 (p‑AKT1/2/3, ~60 kDa), total AKT1/2/3 (~60 kDa), and Vinculin (~116 kDa) used for the quantifications presented in Figure 6d–e from human PASMCs. Molecular weight markers (kDa) are indicated on the left. Vinculin was used as a loading control. Images are shown as exported from the Simple Western system without contrast or intensity adjustments.

**Supplementary Figure 7: Unedited Simple Western images corresponding to Figure 7b-d.**
Uncropped and unprocessed Western blot images showing detection of phosphorylated AKT (p‑AKT1/2/3, ~60 kDa), total AKT (~60 kDa), and Vinculin (~116 kDa) used for the quantifications presented in Figure 6b–d from rat and mouse tissue samples, respectively. Molecular weight markers (kDa) are indicated on the left. Vinculin was used as a loading control. Images are shown as exported from the imaging system without contrast or intensity adjustments.
